# New Insights into Beta-Lactam Resistance of *Streptococcus pneumoniae*: Serine Protease HtrA Degrades Altered Penicillin-Binding Protein 2x

**DOI:** 10.3390/microorganisms9081685

**Published:** 2021-08-08

**Authors:** Katharina Peters, Inga Schweizer, Regine Hakenbeck, Dalia Denapaite

**Affiliations:** Department of Microbiology, University of Kaiserslautern, 67663 Kaiserslautern, Germany; Katharina.Peters@newcastle.ac.uk (K.P.); ingaschweizer@gmx.net (I.S.); hakenb@rhrk.uni-kl.de (R.H.)

**Keywords:** *Streptococcus pneumoniae*, penicillin-binding protein PBP2x, β-lactam resistance, two-component regulatory system CiaRH, serine protease HtrA

## Abstract

Reduced amounts of the essential penicillin-binding protein 2x (PBP2x) were detected in two cefotaxime-resistant *Streptococcus pneumoniae* laboratory mutants C405 and C606. These mutants contain two or four mutations in the penicillin-binding domain of PBP2x, respectively. The transcription of the *pbp2x* gene was not affected in both mutants; thus, the reduced PBP2x amounts were likely due to post-transcriptional regulation. The mutants carry a mutation in the histidine protein kinase gene *ciaH*, resulting in enhanced gene expression mediated by the cognate response regulator CiaR. Deletion of *htrA,* encoding a serine protease regulated by CiaR, or inactivation of HtrA proteolytic activity showed that HtrA is indeed responsible for PBP2x degradation in both mutants, and that this affects β-lactam resistance. Depletion of the PBP2x_C405_ in different genetic backgrounds confirmed that HtrA degrades PBP2x_C405_. A GFP-PBP2x_C405_ fusion protein still localized at the septum in the absence of HtrA. The complementation studies in HtrA deletion strains showed that HtrA can be overexpressed in pneumococcal cells to specific levels, depending on the genetic background. Quantitative Western blotting revealed that the PBP2x amount in C405 strain was less than 20% compared to parental strain, suggesting that PBP2x is an abundant protein in *S. pneumoniae* R6 strain.

## 1. Introduction

Beta-lactam resistance in *Streptococcus pneumoniae* is a multifactorial process that is related mainly to the acquisition of multiple mutations in the transpeptidase domains of penicillin-binding proteins (PBPs), the target enzymes for this class of antibiotics [1]. Such alterations decrease the binding affinity of PBPs to the antibiotic in comparison to their wild type alleles in sensitive strains, and hence growth and division of mutated bacteria proceed even in the presence of the antibiotics [2,3]. *S. pneumoniae* contains five high molecular mass PBPs named PBP1a, PBP1b, PBP2x, PBP2a, and PBP2b, as well as the low-molecular weight PBP3 [4,5]. PBP2x and PBP2b have been confirmed to be essential for cell growth [6,7,8]. Besides being target enzymes for β-lactams, PBPs are also important enzymes involved in the last stages of peptidoglycan biosynthesis, where they play specific roles in cell division and peripheral cell growth (reviewed in [9]).

The main players in the β-lactams resistance process are PBPs 2x, 2b, and 1a (reviewed in [1]). Alterations in all other PBPs have been described occasionally [1,10]. Mutations in PBP2x and PBP2b result in low level β-lactam resistance [11,12,13] and are prerequisite for high level β-lactam resistance mediated by an altered PBP1a [14,15,16]. PBP2x alterations result in low-level resistance to cephalosporins, such as cefotaxime [17], while PBP2b does not interact with cefotaxime and other β-lactams with a similar side chain [18]. Thus, alterations in both PBP2x and PBP2b are necessary for primary penicillin resistance [19,20], and high-level resistance to cefotaxime requires a modified form of PBP1a in addition to modifications in PBP2x [14].

Resistance development differs notably between clinical isolates and laboratory mutants. In resistant clinical isolates, the PBP genes show complex mosaic structure, with sequence fragments of varying lengths that may differ up to 10% at the amino acid level compared to the equivalent regions in PBPs of sensitive pneumococci [11,12,21]. These sequence blocks are acquired from closely related species from Mitis group members of the genus Streptococcus such as *S. mitis* and *S. oralis* through interspecies horizontal gene transfer followed by recombination events [22,23,24,25,26]. In contrast, β-lactam resistant laboratory mutants harbor only point mutations in PBP genes [27]. Depending on the selective antibiotic, different mutational pathways were observed. Laboratory mutants selected with cefotaxime have alterations in PBP2x and PBP2a, rarely in PBP3 [10,28,29], whereas selection with piperacillin results in alterations of PBP2b and in some cases of PBP2x [30]. Remarkably, no mutations in PBP1a of laboratory mutants were obtained.

β-Lactam-resistant laboratory mutants harbor mutations in non-PBP genes, which occur during the selection with β-lactams and are important for the resistance phenotype. In piperacillin resistant mutants, the mutations in the glycosyltransferase CpoA, required for the synthesis of diglycosyldiacylglycerols, resulted not only in decreased susceptibility to piperacillin but were also associated with a reduced amount of PBP1a, reduction of growth rate, and a defect in genetic competence [30,31]. Alterations within the histidine kinase gene *ciaH* of the two-component system (TCS) CiaRH (competence induction and altered cefotaxime susceptibility), have been found in β-lactam resistant laboratory mutants selected with cefotaxime and piperacillin [32]. Every mutant harbors different mutations and thereby had a different *ciaH* allele [33]. These mutations led to enhanced expression levels of genes regulated by the cognate response regulator CiaR and confer two pronounced phenotypes: increased β-lactam resistance and a lack of genetic competence [32,34,35,36]. Furthermore, the lytic behavior of *S. pneumoniae* is also affected by the *ciaH* mutation, and this effect becomes evident especially in comparison with a *ciaR* null mutant [37]. The CiaR regulon is nearly constitutively active under a variety of laboratory conditions [38]. Consequently, the mutations in the kinase-encoding gene *ciaH* lead to hyperactivation of the CiaR regulon [36,39]. 

The CiaRH regulatory system is one of the best studied TCS in *S. pneumoniae* and is involved in the control of different physiological processes (reviewed in [40,41]). The response regulator CiaR directly controls 16 promoters and thereby drives the transcription of 29 genes including five genes specifying small non-coding RNAs [42,43]. These small RNAs, named csRNA for *cia*-dependent small RNAs, are involved in other regulatory networks, including the competence regulon [44]. Another remarkable member of the CiaR regulon is the cell wall-associated serine protease HtrA (high temperature requirement A), which belongs to the HTRA family of proteases that are able act as proteases or chaperones [45]. Members of this family play a key role in protein quality control [46]. The pneumococcal HtrA is associated with a range of physiological processes (reviewed in [41]); however, only a few targets of HtrA have been identified thus far. HtrA plays an important role in the competence at multiple steps by degrading CSP [47] and by removing the DNA uptake apparatus [48]. HtrA also controls bacteriocin activity by degrading the BlpC exporting machinery [49,50] and is critical during heat-induced dispersal of pneumococcal biofilms [51]. Lately, the substantial role of HtrA in bacto-viral co-infections was demonstrated, as well as the significant contribution to pneumococcal colonization [52,53]. Moreover, it has been demonstrated that HtrA targets a GFP-tagged wild type form of PBP2x and derivatives thereof at the septal site [8,54]. Notably, in *S. pneumoniae* HtrA localizes to the equators and septa of most dividing cells [55].

In vitro and in vivo analysis of PBP2x variants from cefotaxime-resistant laboratory mutants revealed clearly reduced amounts of the essential PBP2x in two mutants [56]. In the present study, we examined the molecular basis of this phenomenon by introducing these *pbp2x* alleles in the sensitive laboratory strain *S. pneumoniae* R6. We elucidated the role of the CiaRH regulon in this process focusing on the impact of HtrA. The results of our experiments provide strong evidence that the proteolytic activity of HtrA is responsible for degradation of altered PBP2x, documenting a novel correlation between HtrA and the β-lactam resistance phenotype.

## 2. Materials and Methods

### 2.1. Bacterial Strains, Growth Conditions, and Transformation

Tables S1 and S2 list the bacterial strains and plasmids used in this study. All *S. pneumoniae* strains were derived from R6, a non-encapsulated derivative of the Rockefeller University strain R36A [57]. *S. pneumoniae* strains were grown at 30 °C (in rare cases at 37 °C as indicated) without aeration in C medium [58] supplemented with 0.1% yeast extract (C+Y medium) or on D-agar plates [59] containing 3% (*v*/*v*) defibrinated sheep blood. Pneumococcal cell growth was monitored by nephelometry and is given in nephelometry units (N). For morphological and physiological analysis, cells were inoculated in pre-warmed C+Y medium and incubated at the appropriate temperature. When cell density reached N = 70 (mid-exponential phase of growth), the cells were diluted 1:20 into pre-warmed C+Y medium and growth was followed throughout the growth cycle. The doubling time was calculated using the following formula: µ [min^−1^]$=\frac{\left(lnN-lnN_{0}\right)}{\left(t-t_{0}\right)}$; g [min]$=\frac{ln2}{µ}$ with µ: growth rate; N: Nephelo value at time point t; N_0_: Nephelo value at time point 0; (*t* − *t*_0_): time difference in min; g: doubling time in min. 

To employ a Cheshire cassette [60], we used Todd Hewitt Broth (THB, BD Biosciences, Franklin Lakes, NJ, USA medium supplemented with 0.5% yeast extract (THY medium). For microscopic analysis, cells were grown at 30 °C to ensure full folding of GFP as recommended [61]. For induction of P_Zn_, ZnCl_2_ was added to liquid medium or to agar plates at a final concentration of 0.15 mM. For specific depletion experiments, 0.09 mM ZnCl_2_ was used. PBP2x depletion experiments were performed as described previously [8].

Competent *S. pneumoniae* cells were prepared and transformed as described [37]. When the strains C405 and C606 and their derivatives were transformed, a synthetic competence-inducing peptide 1 (CSP-1) was added at a final concentration of 100 ng/mL, and all transformations steps were carried out at 30 °C. Transformation efficiency was determined using *S. pneumoniae* AmiA9 genomic DNA [62]. When required, the growth media of *S. pneumoniae* was supplemented with a final concentration of 200 μg/mL kanamycin (Kan), 200 μg/mL streptomycin (Str), 2.5 μg/mL tetracycline (Tet), 20 μg/mL spectinomycin (Spc), or 15 μg/mL trimethoprim (Tmp) for selection of transformants.

*Escherichia coli* strain DH5α (Invitrogen) was used as host for propagation of plasmids and for standard cloning procedures, and BL21 (DE3) pLysS (Stratagene, La Jolla, CA, USA) strain was utilized for recombinant protein production. *E. coli* strains were grown aerobically at 37 °C either in Luria–Bertani (LB) medium or on LB agar plates supplemented with ampicillin (100 μg/mL), when required. *E. coli* transformation was performed according to Hanahan [63], and transformants were selected with the appropriate antibiotic. Growth of *E. coli* was monitored by measuring the optical density at 600 nm using a spectrophotometer (Biochrom Ltd., Cambridge, UK).

### 2.2. Microscopy and Cell Length Measurements

For microscopic analysis, 5 μL of exponential growing cells at N = 70 were transferred onto a microscope slide and analyzed using an Eclipse E600 (Nikon, Tokyo, Japan) microscope equipped with a 100 × NA 1.4 oil immersion objective and a DXM1200C camera (Nikon, Tokyo, Japan). The image analysis and determination of the cell size were performed with the Nikon Nis-Elements BR (version 3.2) imaging software or/and ImageJ program (version 1.50). Fluorescence signals of GFP were visualized utilizing the Epi-FL filter block for fluorescence B-2E/C (EX: 465–495, DM: 505, BA: 515–555; Nikon, Tokyo, Japan), thereby using identical fluorescence intensity and exposure times between 1 and 2 s. 

Cell length of parental strain R6, C405, and R6*_2x_*_C405_ growing in C+Y medium at 30 °C were measured from phase-contrast images by using the Nikon Nis-Elements BR software (version 3.2), and the results were further analyzed in Microsoft Excel. More than 100 cells from at least two independent experiments were measured for each strain. Only cells showing a clear invagination at the division septum (late divisional cells) were measured.

### 2.3. Determination of Minimal Inhibitory Concentrations

To determine minimal inhibitory concentrations (MICs), cultures of *S. pneumoniae* were grown in C+Y medium to a cell density of N = 30 and after 1000-fold dilution in 0.9% NaCl, and 30 μL aliquots were spotted on D-agar plates containing the appropriate antibiotic. A very narrow range of antibiotic concentrations was used to visualize minor differences in β-lactam susceptibilities. The gradation of the cefotaxime concentrations was carried out in 0.02 increments (µg/mL). The MIC values were monitored after 24 and 48 h incubation at 37 °C or 30 °C. Mean values from at least three independent experiments were used. D-agar plates were photographed (Sony DFW-X700 camera, Tokyo, Japan), and representative spots were visualized using Adobe Photoshop Elements program (version 2.02).

### 2.4. Determination of β-galactosidase Activity 

The β-galactosidase activity in strains carrying the promotor regions P*_htrA_*, P*_vegM_*, and P*_16S rRNA_* in front of a promoter-less *E. coli lacZ* gene was determined as described previously [64]. The strains were grown in C+Y medium, and β-galactosidase activity was measured throughout the growth of the culture at six time points: when cell density reached N = 30; N = 60; N = 90; N = 120; N = 150; and at the beginning of the stationary phase, more precisely, 30 min after reaching N = 150. For each measurement, the volume of the harvested culture was normalized to contain the equivalent of 2 mL of cells at a cell density of N = 90. The β-galactosidase activities are expressed in nanomoles of nitrophenol released per minute per milligram of protein. The total protein concentrations of the reactions were determined by the method of Bradford [65]. Student’s *t*-test was applied to determine the significance of the results.

### 2.5. RNA Preparation and Quantitative Real-Time PCR

For total RNA isolation, 145 mL of C+Y medium was inoculated with 5 mL of an exponential growing *S. pneumoniae* culture, and the cells were grown to a cell density of N = 70, pelleted at 8000 × rpm at 4 °C, and flash frozen in liquid nitrogen for further use. RNA preparation was performed as described previously [66]. Prior to cDNA synthesis, the RNA was incubated with DNase I (New England Bioloabs, Ipswich, MA, USA) for 15 min at 37 °C and additionally purified using the RNeasy Midi Kit as described by the manufacturer (Qiagen, Düsseldorf, Germany).

Quantitative real-time PCR analyses were performed as described previously [42] and were carried out with the Light Cycler system (Roche, Basel, Switzerland). Each sample was measured in triplicate with three independent biological samples. Specific amplifications were confirmed by single peaks in melting curve analysis. As an unregulated control, the gyrase gene *gyrA* was used for normalization of transcript amounts. Primers used for qRT-PCR are listed in Table S3. Relative quantification was performed using the Light Cycler software 4.0 (Roche). 

### 2.6. Bocillin^TM^FL Labelling of PBPs and Western Blot Analysis

Sample preparation for Western blots, PBP labelling of cell lysates with Bocillin^TM^FL, and separation of proteins by sodium dodecyl sulfate (SDS)-polyacrylamide gel electrophoresis (PAGE) were carried out as described previously [8]. Bocillin^TM^FL–PBP complexes were detected by fluorography with a FluorImager 595 fluorescence scanner (Molecular Dynamics, Chatsworth, CA, USA) at 488 nm. The ImageQuant software (Version 5.3, Molecular Dynamics) was used to document the results.

Western blotting was performed in two different ways: the routine Western blotting as described previously [8], and quantitative Western blotting as described in Section 2.9 of the Materials and Methods. PBP2x and GFP-tagged PBP2x proteins were detected by Western blotting using a 1:10,000 dilution of the primary affinity purified polyclonal anti-PBP2x antibody [8,56], the anti-GFP polyclonal antibody (rabbit IgG fraction; Invitrogen, Waltham, MA, USA) at 1:2000 dilution, or the HtrA polyclonal antibody using a 1:30,000 dilution (see below) followed by incubation with alkaline phosphatase-conjugated goat anti-rabbit immunoglobulin G (Sigma-Aldrich, Burlington, MA, USA) at a 1:10,000 dilution and staining with 4-nitrobluetetrazolium chloride and 5-bromo-4-chloro-3-indolylphosphate (Roche, Basel, Switzerland). It should be underlined that long staining during the detection step of the signals affects the band intensity. To avoid overstaining of the blots and ensure quantitative staining, we quickly stopped the detection reaction, and then the membranes were scanned immediately. Quantitative analysis was performed using the program ImageJ (version 1.50). 

### 2.7. Expression and Purification of the Recombinant Protein HtrA

For recombinant protein production, the plasmid pKP041 was transformed into the *E. coli* strain BL21 (DE3) pLysS. A one-liter culture was grown in LB supplemented with kanamycin (25 µg/mL) to an OD_600_ of 1.0–1.2 at 37 °C. Overexpression of the His-tagged protein was induced with 1 mM IPTG (isopropyl-β-D-1-thiogalactopyranoside), and cultivation was continued for 3–4 h with shaking at 30 °C. All purification steps were performed on ice. Cells were harvested by centrifugation at 8000 × rpm at 4 °C for 15 min and resuspended in 30 mL lysis buffer containing 50 mM Tris, 200 mM NaCl, 2 mM β-mercaptoethanol, and 10 mM imidazole (pH 8.0). Subsequently, cells were lysed by sonication (7 × 2 min on ice) using a Branson sonifier B-12. The lysate was clarified by centrifugation at 21,000× *g* at 4 °C for 40 min. The supernatant was frozen in liquid nitrogen and subsequently stored at −80 °C. 

For purification of His-6-HtrA_S234A_oTM (residues 31–393, the signal sequence was replaced by a thrombin-cleavable N-terminal oligo histidine tag), the supernatant was incubated at 4 °C under continuous stirring for 1 h with 2.5 mL Superflow Ni-NTA agarose (Qiagen, Düsseldorf, Germany), equilibrated with lysis buffer. The agarose was loaded onto a gravity column and washed twice with washing buffer containing 50 mM Tris, 300 mM NaCl, 2 mM β-mercaptoethanol, and 20 mM imidazole (pH 8.0). His-6-HtrA_S234A_oTM was eluted in 6-7 1 mL fractions of elution buffer (50 mM Tris, 300 mM NaCl, 2 mM β-mercaptoethanol, 250 mM imidazole (pH 8.0)). The elution buffer was exchanged with 20 mM sodium phosphate buffer using a desalting column (PD-10 columns, Amersham Biosciences, Buckinghamshire, UK). Aliquots of purified His-6-HtrA_S234A_oTM were flash frozen in liquid nitrogen and stored at −80 °C. The protein concentration was determined by measuring the adsorption at OD_280_ with a spectrophotometer (Nanodrop ND-1000, NanoDrop Technologies, Wilmington, DE, USA) and using the standard Bradford method [65]. The His-tagged protein HtrA_S234A_oTM was separated by SDS-PAGE, and its integrity and purity were analyzed by Coomassie brilliant blue staining and by Western blotting using a 1:1000 dilution of the primary antisera anti-HtrA antibody [67].

### 2.8. HtrA Antisera Production and Immunoblotting

Rabbit polyclonal antiserum against pneumococcal recombinant HtrA was generated by Seqlab GmbH (Göttingen, Germany). No cross-reaction was observed in any of the pre-immune sera with any of the tested *S. pneumoniae* proteins (GST-PBP2x, His6-CiaHE) or with BSA. Rabbit anti-HtrA antisera were used in a dilution of 1:30,000.

### 2.9. Determination of PBP2x Copy Number in S. pneumoniae Using the Quantitative Method of ECL (Enhanced Chemiluminescence)

The cellular amounts of PBP2x protein in *S. pneumoniae* strains R6 and C405 were defined by quantitative Western blotting analysis. Pneumococcal cells were grown in C+Y media at 37 °C to exponential phase (N = 70). A total of 2 mL of culture was collected by centrifugation, and cell pellets were resuspended in 20 mM sodium phosphate buffer, pH 7.2, containing 0.2% (*w*/*v*) Triton X-100. The volume of sample was adjusted as follows: 5 μL of cell suspension corresponded to 2 mL of culture of N = 20. The samples were incubated for 30 min at 37 °C. For blots, 5 μL of cell lysate or/and serial dilutions of the extracts were mixed with 2 × Laemmli SDS loading buffer, and the mixtures were incubated at 95 °C for 5 min. Lysates were centrifuged shortly at 14,000 rpm, and samples were separated in a 10% SDS-PAGE gel. A set of standards with known amounts of purified GST-PBP2x, which contains a soluble PBP2x derivative (residues 49–750), were used as standard in the SDS-PAGE gel. GST-PBP2x was expressed and purified according to the previously described procedure using the plasmid pPM20 [56]. The concentration of GST-PBP2x was determined by the absorbance at 280 nm using a Nanodrop (ND-100, NanoDrop Technologies, Wilmington, DE, USA).

After SDS-PAGE, the proteins were transferred to a nitrocellulose membrane (Protran BA83, Sigma-Aldrich Whatman, Burlington, MA, USA) with a pore size of 0.2 µm and detected with affinity purified polyclonal anti-PBP2x antibody. The secondary goat-anti-rabbit IgG HRP-conjugated antibody (Thermo Scientific, Waltham, MA, USA) was used at a 1:10,000 dilution, and immunoblots were developed by exposure to SuperSignal West Femto substrate (Thermo Scientific) according to the manufacturer protocol. The chemiluminescence signal was detected using the ODYSSEY® Fc imaging system (Dual-Mode Imaging System, Li-COR Biosciences). Quantitative analysis was performed with the LI-COR, Image Studio program (Version 2.0). The determined band intensity of GST-PBP2x (103.93 kDa) was converted, and the linker region and the GST-tag of this construct were deducted by calculating a correction factor. The molecular weight of the soluble PBP2x derivative was defined as 77,520 Da, based on of the amino acid sequence. The values determined in this way, which corresponded to a soluble PBP2x protein, were plotted against the measured PBP2x protein amounts.

The total numbers of *S. pneumoniae* R6 cells were analyzed in liquid media (cell density of N = 70) using a cell counting chamber (Neubauer-improved, Optik Labor, Lancing UK) and an Eclipse E600 (Nikon, Tokyo, Japan) microscope equipped with a 40×/1.4 oil immersion objective. The number of living cells in a culture (CFU) was determined by viable counts on D-agar blood plates as triplicate.

### 2.10. DNA Manipulations and Construction of Mutants

All DNA techniques and the construction of strains (Table S1) and plasmids (Table S2) are described in the Supplementary Materials Section.

## 3. Results

### 3.1. The Cefotaxime-Resistant Laboratory Mutants C405 and C606 Contained Reduced Amounts of PBP2x

**Figure 1:**
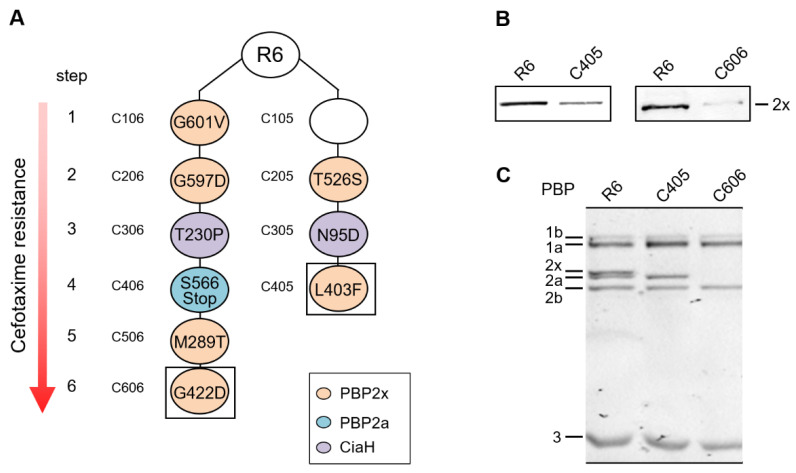
Characteristics of C405 and C606 mutants. (**A**) Cefotaxime-resistant laboratory mutant families C006 and C005 and the occurrence of mutations at each selection step are shown. The first digit of the mutant indicates the selection step, and the last one specifies the mutant family. Mutations identified in PBP2x [19,68], PBP2a [10], and CiaH [36] are shown; the mutation present in C105 is not known. The black squares mark the two mutants C606 and C405, which contain reduced amount of the essential PBP2x. (**B**) Reduced amounts of PBP2x are detected on immunoblot with an anti-PBP2x antibodies. (**C**) PBP profile analysis. PBPs in cell lysates were labeled with Bocillin^TM^FL, and SDS-PAGE gel analyses were performed as described in the Materials and Methods section. The positions of PBPs are indicated on the side. The strain names are indicated on top.

Reduced amounts of essential PBP2x were previously reported in the cefotaxime-resistant mutants C405 and C606 isolated independently from the sensitive laboratory strain *S. pneumoniae* R6. The decrease in PBP2x amounts in C405 and C606 correlates with the introduction of the PBP2x mutations L403F and G422D, respectively [56] (Figure 1). Both mutants also carry different mutations in *ciaH*, resulting in hyperactivation of the response regulator CiaR [36]. In addition, the mutant C606 contains a 119 bp tandem duplication within the PBP2a gene, resulting in a change of amino acid composition after residue 564 and a truncated peptide of 566 aa [10]. In C606 and C405, PBP2x was not detectable after separation by SDS-PAGE and Bocillin^TM^FL labelling (Figure 1C). In addition, in C606 PBP2a could not be detected in the PBP profile on fluorograms, which is in agreement with published data [10].

Western blot analysis of whole-cell extracts using anti-PBP2x antibodies demonstrated that the C405 mutant contained five times less essential PBP2x compared to the R6 strain. In the C606 mutant, the reduced amount of PBP2x was even more pronounced (< 20%) (Figure 1B). Remarkably, mutants C405 and C606 grew to a similar cell density compared to the parental strain R6 at 30 °C and 37 °C. However, they had a slightly increased doubling time compared to R6 (Figure S1). The R6 strain grew with a generation time of 42 min at 30 °C, compared to C606 at 43 min and C405 even at 47 min. Next, the cell morphology of mutants at 30 °C were examined. At mid-exponential growth phase, the C405 cells were smaller (0.92 µm ± 0.03, ± indicates the standard deviation) in comparison to the R6 cells (1.01 µm ± 0.02) (Figure S1). Moreover, evidence was obtained suggesting that C606 is genetically unstable (see Section 3.5).

### 3.2. Determination of PBP2x Copy Number in R6 and C405 

**Figure 2:**
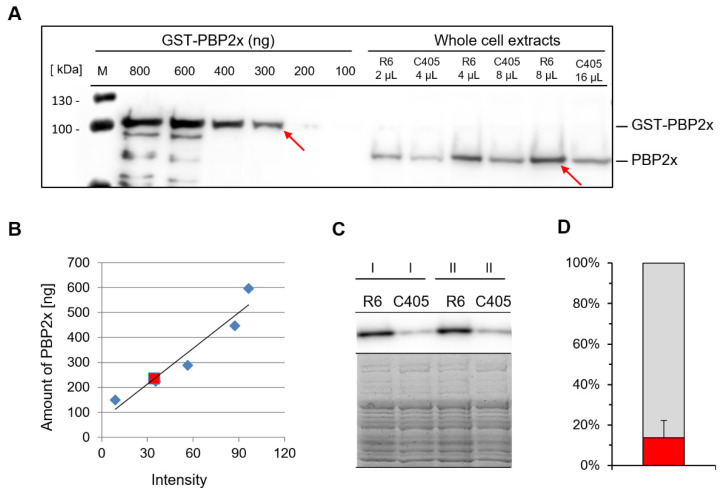
PBP2x is an abundant protein in the parental strain R6. (**A**) Representative quantitative Western blot used to determine the cellular amount of PBP2x. Left lanes 2–7, purified GST-PBP2x standard at various dilutions (800 ng, 600 ng, 400 ng, 300 ng, 200 ng, 100 ng, respectively); right lines (8–13) native PBP2x detected in different amounts of whole-cell lysates of R6 and C405 strains. M, molecular weight standard. The ODYSSEY® Fc imaging system was used to detect the chemiluminescence signal. Red arrows mark bands with identical band intensities. (**B**) Standard curve generated from (**A**). The red square marks the chemiluminescence of the PBP2x band from 8 μL of total R6 cell lysate, and the intensity of the signal (34.4) corresponded to 235.7 ng of soluble PBP2x protein. (**C**) Quantitative determination of PBP2x protein in the mutant C405 compared to the parental strain R6. Equal amounts from two independent cell extracts (I and II) were separated by 10% SDS-PAGE, and the densities of PBP2x bands were detected by quantitative Western blotting using ECL (top). The presence of equal amounts of protein in the loaded samples was verified by Coomassie blue staining after separation by SDS-PAGE (bottom). (**D**) The difference of PBP2x amounts between the two strains R6 (in gray, 100%) and C405 (in red, 15.9%) is shown. The mean values of determined protein amounts (%) from five independent experiments are visualized.

We determined the amount of cellular PBP2x in the parental strain R6 by quantitative Western blotting using affinity purified anti-PBP2x antibodies, as described in the Materials and Methods section. Various concentrations of purified soluble GST-PBP2x were used as standard. The quantification of PBP2x protein in exponentially growing R6 cells is shown in Figure 2A. The amount of PBP2x detected in R6 was within the linear range of the standard curve (Figure 2B). The cell number of R6 was determined by two different methods. The cell number counted in liquid media resulted in 225,600 cells/µL, whereas by viable counts, the number was 176,300 cells/µL. Consequently, the average number of PBP2x molecules per cell in R6 was approximately 20,000, indicating that PBP2x is an abundant protein in *S. pneumoniae.*

Given the fact that both mutant strains C405 and C606 contained reduced amounts of the essential PBP2x whereas the growth and division process was not significantly impaired, we quantified the PBP2x amounts in C405 and R6 by quantitative Western blotting (Figure 2C). The representative results of five independent experiments are depicted in Figure 2D. The PBP2x amount in C405 strain was less than 16% (which corresponded to a 6.3-fold reduction) compared to that in R6. Taken together, these results suggest that at least wild type cells contain an apparent surplus of PBP2x proteins.

### 3.3. Introduction of Altered PBP2x Alleles into R6 Background Also Led to Reduced Protein Amount 

**Figure 3:**
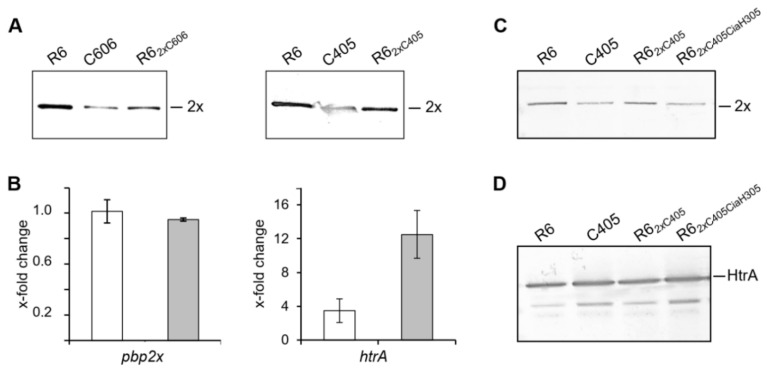
Impact of PBP2x mutations and hyperactivated CiaRH system on PBP2x amounts in the R6 background. (**A**) The reduced amounts of PBP2x are visualized on Western blots using anti-PBP2x antibodies. (**B**) Transcription of *pbp2x* (left) and *htrA* (right) in mutants C405 (white) and C606 (gray) were measured by RT-PCR. Results are shown as fold changes of *pbp2x* or *htrA* expression relative to the parental strain R6. Western blot analysis using anti-PBP2x (**C**) and anti-HtrA (**D**) antibodies. The position of PBP2x or HtrA are indicated on the left side. Strains used for cell lysate preparation are indicated on top.

To test if the PBP2x mutations by themselves are responsible for the reduced amount of the protein, we introduced the two *pbp2x* alleles into the parental strain *S. pneumoniae* R6 by transformation and selection with low concentrations of cefotaxime [56]. In both constructs, the amount of PBP2x was higher compared to that of the original mutants but reduced compared to R6 (55–75%) (Figure 3A), documenting that other factors are responsible for the decrease detected in the mutants C405 and C606. Notably, the same results were produced independently of the growth temperature (data not shown). The transformant R6*_2x_*_C606_ had longer doubling times (47 min, compared to parental strain R6 (42 min)), and the cells of R6*_2x_*_C405_ at 30 °C were slightly longer (1.18 μm ± 0.02, *n* = 86) in comparison to parental strain R6 (1.01 μm ± 0.02, *n* = 180) (Figure S2).

### 3.4. Transcription of Pbp2x

To see whether the reduced amounts of PBP2x in the mutant strains were due to changes at the transcriptional level, we used RT-PCR to quantify the expression levels of *pbp2x.* In both mutant strains, the *pbp2x* expression was indistinguishable from that in the parental strain R6 (Figure 3B, left) suggesting the post-transcriptional regulation. One possible reason could be that the altered PBP2x proteins in C405 and C606 strains are either unstable or directly degraded by a protease.

### 3.5. PBP2x and the Hyperactivated CiaRH System 

Both mutants C405 and C606 carry different *ciaH* mutations, resulting in enhanced CiaR-mediated gene expression. The *ciaH305* allele stimulates the transcription of CiaR-dependent promoters 4-fold, and the *ciaH306* allele 10-fold [36]. To assess the influence of *ciaH* alleles individually on the altered PBP2x protein, we introduced the *ciaH305* and the *ciaH306* alleles into R6*_2x_*_C405_ and R6*_2x_*_C606_, respectively, using the Janus counterselection procedure [69], and the amount of PBP2x mutant protein was determined (Figure 3C and Figure S3). Western blotting with anti-PBP2x antibodies showed that the introduction of the *ciaH* alleles affected the amount of PBP2x mutant protein. Thus, the combination of mutations in genes *pbp2x* and *ciaH* was responsible for the reduced PBP2x amounts in C405 and C606, and mutations in other genes are apparently not involved in this phenomenon. It should be noted that the mutant R6*_2x_*_C606_ carrying *ciaH306* allele was genetically unstable, as well as the strain C606, and therefore the presence of the expected mutations was verified by sequencing in all experiments. Frequently, complementary mutations in CiaH or CiaR genes were observed in the mutant strains, which directly affected the amount of PBP2x (Figure S3). This clearly suggests that strong upregulation of the CiaRH system is not well tolerated in pneumococcal cells. In further experiments, we focused on C405 and R6*_2x_*_C405_ derivatives. 

The gene encoding the serine protease HtrA is strongly regulated by CiaR [38,70] and has been shown to target a GFP-tagged wild-type PBP2x and derivatives thereof [8,54]. Thus, the expression of *htrA* was analyzed by RT-PCR in mutants C405 and C606 in comparison to the parental strain R6. The *htrA* expression level was 3.5-fold higher in C405 and 12.5-fold higher in C606 compared to the parental strain R6 (Figure 3B, right). These results are in line with promoter activities of *htrA* measured in R6 strains carrying *ciaH305* and *ciaH306* alleles [36]. The protein level of HtrA in R6 and the mutants was determined by Western blotting using HtrA-specific antibodies (Figure 3D). We detected smaller, immunoreactive bands, most probably representing HtrA degradation products in C405 or R6*_2x_*_C405CiaH305_, which were stronger compared to R6.

### 3.6. The Serine Protease HtrA Degraded the Essential Altered PBP2x in the Cefotaxime-Resistant Mutants C405 and C606

**Figure 4:**
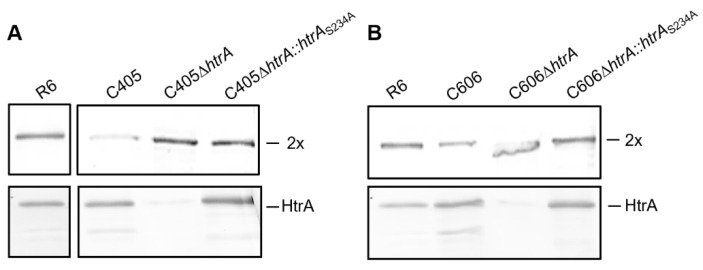
Deletion of HtrA or inactivation of its proteolytic activity in C405 (**A**) and C606 (**B**). Western blots using anti-PBP2x antibodies (upper panel) and anti-HtrA antibodies (lower panel). The strains are indicated on top. Positions of PBP2x and HtrA are shown. Note the increased degradation products of HtrA in C405 and C606 strains (protein band detected below the HtrA protein band).

Consequently, we examined if HtrA targets PBP2x_C405_ and PBP2x_C606_ proteins. We investigated two different constructs. In the first construct, *htrA* was deleted using a kanamycin resistant gene *aphIII*. In the second, the deletion was complemented with an HtrA derivative containing a mutation S234A in the catalytic active site, resulting in a protease inactive derivative. In C405 and C606, the deletion of *htrA* or its inactivation at the catalytic site (*htrA_S234A_*) restored the amount of altered PBP2x compared to parental strain R6 (shown by Western blotting using anti-PBP2x antibodies, Figure 4). The data also confirmed that HtrA was clearly overexpressed in C405 and C606 mutants (Figure 4). 

### 3.7. Complementation Studies in HtrA Deletion Strains

Furthermore, we investigated whether HtrA is responsible for the reduced PBP2x amounts seen in the original mutants and also in the R6*_2x_*_C405_ and R6*_2x_*_C606_ mutants. The HtrA gene was deleted in both mutant strains and in the parental strain R6 using the Cheshire cassette, as described in the Supplementary Materials Section. Then, the HtrA deletion strains were complemented to achieve ectopic expression of *htrA* gene under the control of different promoters, which vary in strength to determine if the amount of altered PBP2x correlates with an increased expression of HtrA. Using this approach, the expression of *htrA* could be investigated independent of the CiaRH system. For this purpose, the anhydrotetracycline inducible promoter P_xyl/tet_ [39] was used, but the expression level of *htrA* did not exceed that of the parental strain R6. Furthermore, high levels of expression were not reached when fully induced using different inducible conditions (data not shown). Next, we tested three different promoters: the native promoter (P*_htrA_*), the constitutive expressed *vegM* (P*_vegM_*) promoter, and the very strong constitutive promoter from 16S rRNA (P*_16S rRNA_*). The latter promoter was constructed as described in the Supplementary Materials (Figure S4).

**Figure 5:**
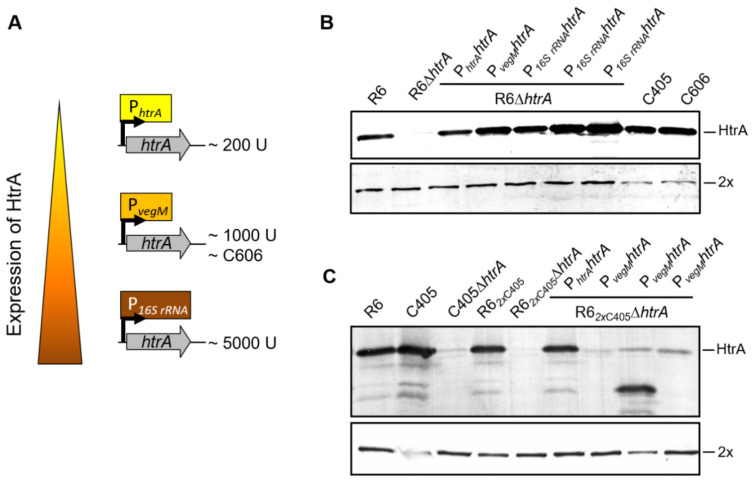
Complementation of HtrA deletion strains. (**A**) The HtrA gene under the control of different promoters was introduced next to the *bgaA* gene into the genome of HtrA deletion strains (Figure S5). Complementation of HtrA deletions strains in R6 (**B**) and in R6*_2x_*_C405_ (**C**) background. Western blots were developed with anti-HtrA (top) and anti-PBP2x (bottom) antibodies. Positions of HtrA and PBP2x are indicated. Strains used for cell lysate preparation are indicated on top. Three independent transformants carrying either *htrA* under the control of P*_16S rRNA_*(**B**) or P*_vegM_* (**C**) promoter region are shown.

First, we evaluated the promoter activities using a promoterless *E. coli lacZ* as the reporter gene and a stable integration of promoter-*lacZ* fusions driven by the promoter probe vector pPP2 [64]. The β-galactosidase activities were measured at six time points taken throughout the growth of the culture (Figure S5). The promoter activities are illustrated in Figure 5A. P*_htrA_* belongs to moderate expression promoters (≈200 U). The constitutive promoter P*_vegM_*was found to be a strong promoter, resulting in a similar expression level of *htrA* gene as in C606 (≈1000 U), whereas the P*_16S rRNA_* promoter was five times stronger than the P*_vegM_* promoter.

Second, the expression of *htrA* gene was placed under the control of these three promoters. The constructs were cloned into the integration plasmid pSW1 (Table S2). It replicates in *E. coli* and integrates into the genome of *S. pneumoniae* at the *bgaA* site by homologous recombination, thereby replacing an intergenic region between *bgaA* and the adjacent gene *spr0566.* The trimethoprim resistance gene was used to select transformants in *S. pneumoniae* (Figure S6). Three to five independent transformants from each transformation were verified by PCR and sequencing. Western blot analysis demonstrated that it was not possible to overexpress *htrA* using the very strong 16S rRNA promoter in the R6 parental strain. The transformants carried either different mutations in the P*_16S rRNA_* promoter region, which suppress the expression of HtrA in R6 strain, or mutations in the protease domain, thereby affecting proteolytic activity of HtrA. The *htrA* expression under the control of P*_vegM_* led to increased amount of HtrA protein (like the C405 strain), but the amount of PBP2x in R6 derivatives was not affected (Figure 5B). 

The complementation experiments in R6*_2x_*_C405_ and R6*_2x_*_C606_ mutants demonstrated that the overexpression of *htrA* under the control of P*_vegM_* promoter resulted in different mutations in *htrA* or the promoter region, similar to what was observed for the P*_16S rRNA_* promoter in R6 background. Figure 5C shows an example of the complementation studies in the background of an altered *pbp2x*_C405_ gene. The complementation of HtrA under the control of the native promoter led to reduced amount of PBP2x, verifying the results in Figure 3. The mutants carrying *htrA* under the control of P*_vegM_* promoter had different mutations in *htrA,* which resulted in a truncated version of the HtrA protein or in loss of its protease activity. Surprisingly, the KPKL322 mutant carrying several mutations in HtrA was still able to affect the amounts of PBP2x (Figure 5C, line 8).

Taken together, the results clearly show the overexpression of HtrA in *S. pneumoniae* is only possible to a certain level or not at all, depending on the genetic background. This finding strongly suggests that too much active HtrA is damaging to the cell. Once the tolerated level is exceeded, the cell defends itself with severe mutations that inhibit either the proteolytic activity and/or the synthesis of the serine protease HtrA.

### 3.8. Impact of HtrA on PBP2x-Mediated Cefotaxime Resistance

**Figure 6:**
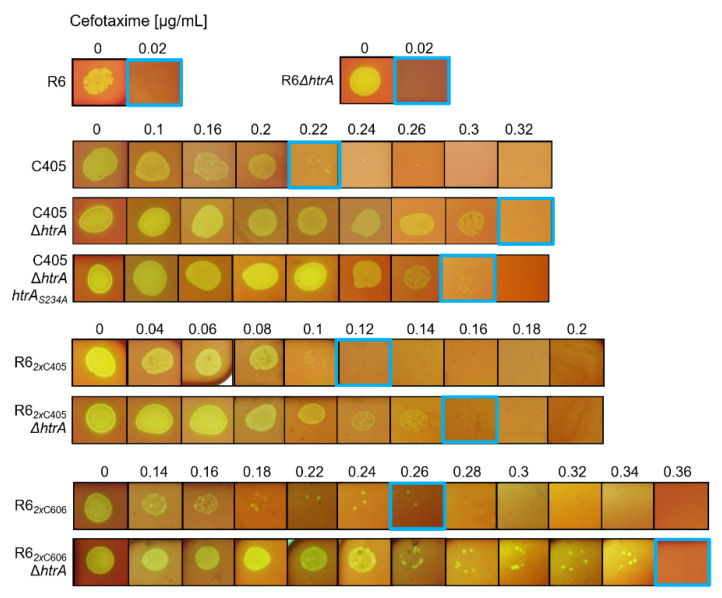
Effect of HtrA on PBP2x-mediated cefotaxime resistance. *S. pneumoniae* strains were grown in C+Y medium to a cell density of N 30, diluted, and spotted on D-blood agar plates containing narrow concentrations of cefotaxime (0.02 (µg/mL) steps). The plates were incubated for 48 h at 37 °C. A representative result of five independent experiments is shown. The spots in blue frame correspond to the defined MIC values.

Deletion of *htrA* in PBP2x mutant strains results in increased amounts of altered PBP2x protein, which is responsible for cefotaxime resistance in the cell. Consequently, we tested whether the presence or absence of HtrA influences PBP2x-mediated cefotaxime resistance. The MIC for cefotaxime was determined on D-agar plates containing a narrow range of increasing concentrations of the antibiotic. As shown in Figure 6, a clear correlation between the absence of HtrA and increased level of cefotaxime susceptibility became apparent. The deletion of HtrA in C405 strain led to a significant increase in resistance. The same correlation was observed by testing the MIC of HtrA deletions strains in the R6*_2x_*_C405_ and the R6*_2x_*_C606_ background. The MIC value of the protease inactive complementation mutant (C405 ∆*htrA htrA*_S234A_) was slightly below that of the *htrA* deletion strain but higher than in C405. Taken together, our data verified the assumptions that HtrA affects PBP2x-mediated cefotaxime resistance and that more mutated PBP2x protein present in the cell confers an even higher level of cefotaxime resistance. 

### 3.9. Localization of Low-Affinity GFP-PBP2x_C405_ Fusion Protein Was Affected by HtrA

**Figure 7:**
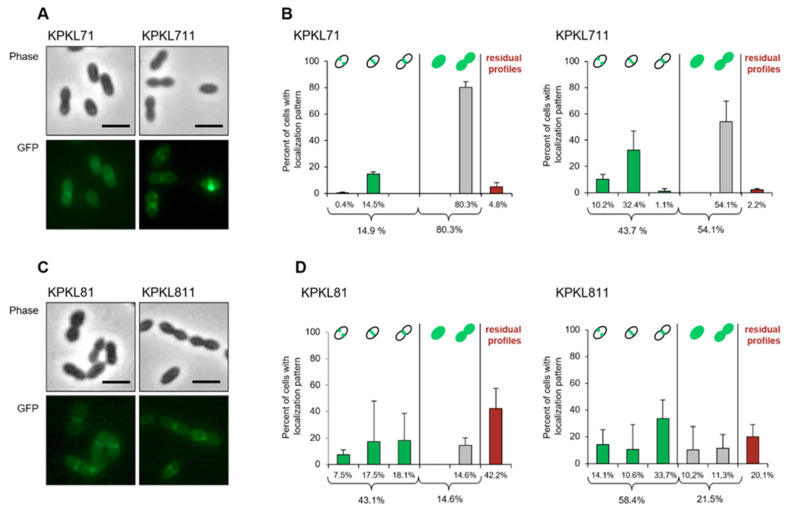
Cellular localization of the GFP-PBP2x_C405_ fusion protein in strains C405 (**A**,**B**) and R6*_2x_*_C405_ (**C**,**D**). (**A**,**C**) Localization of GFP-PBP2x_C405_ in the presence or absence of HtrA. Top: phase contrast microscopy; bottom: fluorescence signal. Scale bar = 2 μm. (**B**,**D**) Classification of cells according to GFP-PBP2x_C405_ localization and the cell division stage (pre-divisional, early divisional, and late divisional) as shown schematically on top. Number of cells analyzed: KPKL71, *n* = 899; KPKL711, *n* = 379; KPKL81, *n* = 1161; KPKL811, *n* = 637. Cells were grown in C+Y medium in the presence of ZnCl_2_, and microscopy images were performed as described in the Materials and Methods. Strains had the following genotype (Table S1): KPKL71 (C405 *bgaA*::P_Zn_*-gfp-2x*_C405_ ∆*2x*_C405_), KPKL711 (C405 *bgaA*::P_Zn_*-gfp-2x*_C405_ ∆*2x*_C405_ ∆*htrA*), KPKL81 (R6*_2x_*_C405_ *bgaA*::P_Zn_*-gfp-2x*_C405_ ∆*2x*_C405_), KPKL811 (R6*_2x_*_C405_ *bgaA*::P_Zn_*-gfp-2x*_C405_ ∆2x_C405_ ∆*htrA*).

The effect of the reduced amount of PBP2x on the localization of the mutated and low-affinity PBP2x variant from C405 was investigated by fluorescence microscopy (Figure 7). We generated an N-terminal GFP-PBP2x_C405_ fusion protein using the vector pJWV25 and introduced it into the *bgaA* locus of two strains C405 and R6*_2xC_*_405_. This gave rise to strains KPKL7 and KPKL8. Subsequently, the genomic *pbp2x*_C405_ gene was then replaced by a spectinomycin resistance cassette in the presence of zinc, resulting in conditional mutants as described [8]. The strains were named KPKL71 and KPKL81. Western blot analysis of whole-cell extracts using anti-PBP2x antibodies demonstrated the production of full-length GFP-PBP2x_C405_ in the four constructed strains and absence of PBP2x degradation (Figure S7A). In merodiploid strain KPKL8, the fusion protein GFP-PBP2x_C405_ was slightly overproduced (less than twofold). The amount of GFP-PBP2x_C405_ was clearly reduced in C405 derivatives in comparison with R6*_2x_*_C405_ strain. This was also verified by Western blot using anti-GFP polyclonal antibodies, showing the presence of fusion protein under induced conditions and obvious degradation products (Figure S7B). Then, the HtrA gene was deleted in conditional mutants, giving rise to KPKL811 and KPKL711 strains. The amount of GFP-PBP2x_C405_ was clearly higher in the absence of HtrA in both strains, and degradation products were substantially reduced (Figure S7C). 

In most of the KPKL71 cells (80.3%), the fluorescence signal of GFP-PBP2x_C405_ was detected in the cytoplasm, and only a small fraction of KPKL71 cells (14.9%) showed a clear septal localization (Figure 7). The amount of septal localization of GFP-PBP2x_C405_ fusion protein in KPKL81 was much higher (43.1%). The deletion of the serine protease HtrA gene (KPKL711 and KPKL811) correlated with the fact that the fusion protein GFP-PBP2x_C405_ was no longer degraded, resulting in a correct septal or equatorial localization. Thus, the small amount of GFP-PBP2x_C405_ is always located in the region where it is most urgently needed—at the cell septum. In our previous study, we revealed that HtrA targets the functional full-length GFP-PBP2x fusion [8]. These experiments verified that HtrA also targets GFP-PBP2x_C405_ allele that is structurally distinct from the wild-type PBP2x.

### 3.10. Effect of HtrA Deletion in Depletion Studies of Altered PBP2x

Next, we wanted to confirm whether untagged altered PBP2x_C405_ is an HtrA substrate by another method, taking the function of the protein into account rather than detection by Western blot. Therefore, we performed depletion experiments enabling the visualization of small differences in PBP2x amounts on growth, as described previously [8]. In two different genetic backgrounds, C405 and R6_2xC405_, we first inserted an extra copy of the *pbp2x*_C405_ gene into the genome under the control of P_Zn_ inducible promoter using pFP14 plasmid. Next, the native PBP2x_C405_ gene was replaced by a spectinomycin resistance cassette in the presence of Zn^2+^, resulting in the conditional mutant. Subsequently, *htrA* was deleted in these strains.

**Figure 8:**
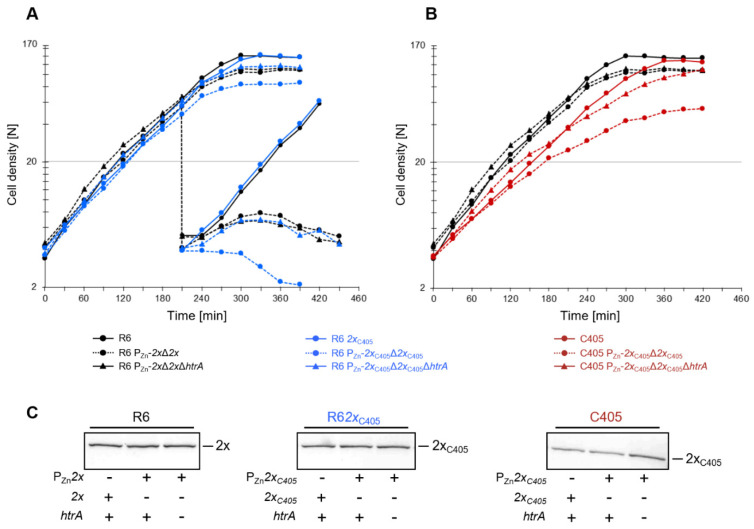
HtrA targets altered PBP2x. Effect of *htrA* deletion on PBP2x_C405_ in (**A**) R6*_2x_*_C405_ (in blue) and (**B**) C405 (in red) backgrounds. R6, DKL41 (R6 P_Zn_-*2x*Δ*2x*), and DKL411 (R6 P_Zn_-*2x*Δ*2x*Δ*htrA*) were used as controls (in black). Strains were grown in the presence of the 0.09 mM ZnCl_2_, pelleted, washed, and diluted 1:20 in C+Y medium without ZnCl_2_. When cell density reached N = 70, the cells were diluted 1:20 (vertical dashed line), except for DKL61 (C405 P_Zn_-*2x*_C405_Δ*2x*_C405_) and DKL611 (C405 P_Zn_-*2x*_C405_Δ*2x*_C405_Δ*htrA*) due to poor growth under these conditions. Further used strains: DKL51 (R6 P_Zn_-2x_C405_Δ*2x*_C405_) and DKL511 (DKL51Δ*htrA*). (**C**) Cells were harvested at mid-exponential growth for Western blot analysis using anti-PBP2x antibodies. The position of PBP2x or PBP2xC405 is indicated. All experiments were repeated at least 3 times with almost identical results.

For PBP2x depletion assays, cells were first grown exponentially in C+Y medium containing 0.09 mM ZnCl_2_. Next, the cultures were pelleted, washed, and diluted in C+Y medium without additional ZnCl_2_ (Figure 8). R6 and R6_2xC405_ derivatives continued to grow equally well in the absence of zinc (Figure 8A), and in both cases PBP2x was present in similar amounts (Figure 8C). After five generations (210 min), slight growth differences between DKL51 (R6 P_Zn_-*2x*_C405_Δ*2x*_C405_) and DKL511 (DKL51 Δ*htrA*) became visible and were even more pronounced at the end of the exponential growth phase. To enforce complete depletion of PBP2x_C405_, we diluted the cells a second time when the culture reached a cell density of N = 70. Only the DKL51 derivative containing the *pbp2x*_C405_ allele stopped growing immediately, followed by cell lysis, whereas DKL511 and control strains still showed some increase in cell density before slow lysis commenced, independent of the *pbp2x* allele (Figure 8A). This also confirms that, in the R6 background, PBP2x_C405_ is present in lower amounts compared to the wild type allele, and that HtrA targets PBP2x_C405_.

Depletion experiments carried out with derivatives of C405 strain (DKL61 and DKL611) showed clear growth difference (Figure 8B). The DKL61 (C405 P_Zn_-*2x*_C405_Δ*2x*_C405_) strain grows slowly and only reaches a cell density of N~60, whereas strain DKL611 (DKL61Δ*htrA*) reaches almost the same cell density as the parental C405 strain. This is consistent with data obtained by Western blot experiments, where DKL611 restores the amount of altered PBP2x compared to parental strain (Figure 8C). Depletion studies of the PBP2x_C405_ in different genetic backgrounds and in presence or absence of HtrA confirmed that HtrA degrades untagged PBP2x in C405.

Altogether, our results suggest that HtrA targets not only GFP-tagged PBP2x variants but also altered PBP2x, thereby affecting PBP-mediated cefotaxime resistance. Remarkably, PBP2x appears to be an abundant protein in pneumococcal cells, and only less than 20% of PBP2x is needed to sustain a normal growth rate in *S. pneumoniae*.

## 4. Discussion

The present study continues investigations of the major β-lactam resistance determinant PBP2x in cefotaxime-resistant laboratory mutants and elucidates the role of the cell wall associated serine protease HtrA, a notable member of the CiaRH regulon [42]. In fact, mutations in *ciaH* were identified in all cefotaxime-resistant laboratory mutants, suggesting that some component of the CiaRH regulon plays a role during resistance development in laboratory conditions. Earlier work showed that the introduction of PBP2x mutations L403F in C405 and G422D in C606 leads to reduced amounts of the essential PBP2x in both mutants [56]. The respective mutations are in the transpeptidase domain of PBP2x but show a completely different pattern in the three-dimensional context and lead to different consequences. G422D is located on the surface of the transpeptidase domain with its side chain directed toward the C-terminal domain, and consequently does not affect the active site directly [71,72]. In agreement with this notion, in vitro experiments using purified PBP2x derivatives showed that the G422D mutation has no significant effect on cefotaxime resistance [68] or on Bocillin^TM^FL binding to the active site [71]. In case of PBP2x_C405_, the L403F mutation is situated very close to the active site on α-helix 5 [56,72]. The introduction of L403F in PBP2x_C405_ leads in the loss of PBP2x detectability on a fluorogram (Figure 1) [68], indicating that the interaction with the β-lactam is practically abolished in PBP2x_C405_. Furthermore, it has been shown that the appearance of both mutations confers an increase in cefotaxime resistance only at 30 °C, whereas an apparent hypersensitivity to cefotaxime was observed at 37 °C [19]. Despite the different location of these two mutations, both affect the stability of the protein itself. 

We now demonstrate that these changes in the PBP2x structure are recognized by HtrA and thus lead to partial degradation of the protein independent of the growth temperature (Figure 1). We could show that the proteolytic activity of HtrA is responsible for PBP2x degradation in the C405 and C606 mutants (Figure 4). Deletion of *htrA* or insertion of a mutation in the active site (S234A) of HtrA led to wild-type amounts of PBP2x in both laboratory mutants C405 and C606. Transformation of the *pbp2x*_C405_ *and pbpb2x*_C606_ alleles into the sensitive parental R6 strain where the CiaRH system is less active as in the mutants resulted in a slight reduction of PBP2x amount, but not as evident as in the mutants (Figure 3A). In a further step, the presence of the *ciaH305* allele in R6*_2x_*_C405_ resulted in equally high amounts of HtrA and reduced amounts of PBP2x, as seen in the mutant C405, clearly showing that HtrA is exclusively responsible for the degradation of PBP2x_C405_. Investigations of the combining effect of *pbp2x*_C606_ with the *ciaH306* allele on PBP2x_C606_ amounts failed due to the genetic instability of the *ciaHC606* allele, as described previously [34,36], resulting in missense mutations within the *ciaRH* operon. Interestingly, two strains (KPKL1011 and KPKL1012) that have gained an additional mutation E255K in *ciaH* (Figure S3) showed similar PBP2x amounts as R6. Possibly, the E255K mutation represses the strong activation of CiaR-dependent gene regulation, resulting in wild-type expression levels, indicating that at least some CiaR-regulated components are potentially deleterious to the cell. To confirm this hypothesis, the promoter activity of *htrA* should be compared in these strains harboring strongly activated *ciaH* alleles, but their genetic instability as well as the fact that the secondary mutations in *ciaR* and *ciaH* were frequently observed in these strains [34,36] pose considerable problems. These secondary mutations result in reduction of CiaRH-mediated gene expression accompanied by decreased cefotaxime resistance, restoration of transformability, and higher growth rates [36]. Our experiments confirm again that strong upregulation of the CiaRH system as described in laboratory mutants is not well tolerated in pneumococcal cells. In contrast, CiaH mutations occur rarely in clinical isolates and lead to an only moderate activation of CiaR, which could help to survive under the adverse conditions of antibiotic treatment, as shown for strain Tupelo [73].

Within comprehensive complementation studies, *htrA* expression was increased stepwise in *htrA* deletion strains R6*_2x_*_C405_ and R6*_2x_*_C606_, correlating with increasing amounts of HtrA and decreasing amounts of PBP2x (Figure 5). These experiments not only verified that HtrA is responsible for degradation of PBP2x variants but also clearly show that HtrA can only be overexpressed in pneumococcal cells up to a certain level or not at all, depending on the genetic background. Once a certain level is exceeded, the cell defends itself with complementing mutations that affect either the proteolytic activity of HtrA or the synthesis of HtrA itself when located in the HtrA promoter region. In the R6 background, HtrA overexpression could be achieved using the *vegM* promoter, which leads to similar high expression levels of *htrA* as in C606 (Figure 5), but higher expression levels as driven by P*_16S rRNA_* was not possible. In contrast, attempts to construct P*_vegM_*-*htrA* complementation strains in R6*_2x_*_C405_ and R6*_2x_*_C606_ resulted in additional mutations in the HtrA gene or in its promoter region. Thus, R6*_2x_*_C405_ and R6*_2x_*_C606_ tolerate the overexpression of HtrA even less than the parental R6 strain, probably because the mutated PBP2x proteins are better protein substrate for HtrA than the wild-type protein. Taken together, all these results indicate that there is a surplus of PBP2x in the cell, and that its degradation by HtrA can only be tolerated up to a certain level. Furthermore, it should be noted that the serine protease HtrA certainly has other targets besides PBP2x [47,48,50], the degradation of which may also have serious consequences for the cell in case of HtrA overexpression. Whether HtrA is the only factor causing the genetic instability of strains harboring strongly activated *ciaH* alleles remains to be determined. 

In a previous study, we showed that pneumococcal HtrA degrades GFP-PBP2x fusion proteins and derivatives thereof, but not native PBP2x [8,54]. To detect even small differences between mutant and wild-type PBP2x as HtrA substrates, we performed depletion assays, since the ability to grow is a very sensitive parameter to determine whether sufficient amounts of an essential protein are present in the cell. First, depletion of the PBP2x_C405_ variant in different genetic backgrounds and in the presence or absence of HtrA confirmed that HtrA degrades altered PBP2x_C405_ protein (Figure 8). Second, the depletion of PBP2x_C405_ protein in C405 was more pronounced as in R6*_2x_*_C405_ strain, in agreement with data obtained on Western blots (Figure 3) and complementation studies (Figure 5). The differences between mutant and parental strain were obvious in GFP-PBP2x_C405_ localization studies as well (Figure 7). We could detect very little degradation of GFP-PBP2x_C405_ fusion protein in R6*_2x_*_C405_, whereas substantial degradation products were observed of GFP-PBP2x_C405_ in the C405 background (Figure S7), in line with high cytoplasmic fluorescent signals dispersed throughout the cell (Figure 7). As soon as the serine protease HtrA was absent in these strains, the fusion protein was no longer degraded and a correct septal or equatorial localization of PBP2x_C405_ was detected. It should be noted that almost 60% of KPKL811 cells where *htrA* was deleted (Figure 7) showed fluorescence signals at the septum (which was, however, still lower compared to 85% GFP-PBP2x fusion protein described in the R6 strain by Peters et al. [8]). Remarkably, pneumococcal HtrA also maintains septal and equatorial localization in most of dividing cells, but the molecular basis for HtrA localization to cell division sites remains to be discovered [55]. Tsui et al. proposed that HtrA might play a general role in the quality control of proteins linked to Sec translocase export pathway [55]. Interestingly, the functional relationship between HtrA and SecA has also been suggested in *S. pyogenes* [74] and more recently *Helicobacter pylori* [75]. 

PBP2x_C405_ confers considerable resistance to cefotaxime with MIC values of 0.12 μg/mL in R6*_2x_*_C405_ compared to 0.22 μg/mL in C405 (Figure 6), whereas the *ciaH305* allele, when introduced into R6, had almost no effect (0.03 μg/mL compared to 0.025 μg/mL in the R6 strain) [36]. We investigated whether the amount of altered PBP2x_C405_ in the cell can contribute to higher level of cefotaxime resistance. Remarkably, the MIC values increased in strains in which *htrA* was deleted (Figure 6). The MIC value of the protease inactive derivative (C405 ∆*htrA htrA*_S234A_) was above the MIC of C405, but slightly below that of the *htrA* deletion strain. This could be an effect of the putative chaperone activity of HtrA (which has never been proven experimentally in *S. pneumoniae*), since HtrA can function as a chaperone and participate in folding reactions [76]. 

What are the consequences of PBP2x mutations for cell division and elongation *in S. pneumoniae*? The R6*_2xC_*_405_ strain with two mutations in *pbp2x* grows in a slightly elongated manner but enlarged or lemon-shaped cells with pointed, conical ends were not observed (Figure S2). A similar effect was observed in PBP2x depletion assay at the time point when depletion of PBP2x was first observed [8] (p. 738, Figure 2B, time point 2). The formation of elongated pneumococcal cells was also reported for the strain D39 ∆*cps* when PBP2x activity was inhibited by methicillin [77,78]. The authors showed that addition of 0.1 μg mL^−1^ methicillin preferentially inhibited >80% of PBP2x transpeptidase activity and caused cell elongation [77], suggesting blocked septal PG synthesis and ring closure under these conditions [78]. In contrast, the cells of C405 were smaller compared to R6 cells (Figure S1). C405 also carries the mutation in *ciaH*, leading to hyperactivation of the CiaR regulon. It is possible that hyperactivation of CiaH in the mutant affects cell morphology and/or pneumococcal cell division and elongation machinery. The interplay between the sensor histidine protein kinase CiaH and penicillin-binding proteins PBP1a and PBP2x has been suggested previously [79].

PBP2x is not only the β-lactam target and primary resistance determinant but is also an essential enzyme involved late stages of peptidoglycan biosynthesis, where it plays a fundamental role in septal PG synthesis [1,9]. It should be emphasized that reduced amounts of PBP2x have been noted only in two cases, C405 and C606, among the laboratory mutants generated independently of the β-lactam used for selection [27]. Thus, no deleterious effect of PBP2x mutations became apparent in the other mutant. This is also seen in the penicillin-resistant clinical isolates of *S. pneumoniae* where the production of low affinity PBPs is the product of mosaic genes generated by horizontal gene transfer [80,81]. In other words, PBP2x variants in clinical isolates are constructed in such a way that they are not targeted by HtrA, thus ensuring proper growth. Nevertheless, the mutant C405 can grow with 6.3 times less PBP2x compared to the R6 strain, and C606 contained not even 16% of this essential protein. Therefore, we determined the amount of cellular PBP2x in R6. Our studies revealed that PBP2x is an abundant protein with approximately 20,000 molecules per cell (Figure 2). This value agrees with another study [82] but differs largely from another report [83] where only 260 ± 60 molecules per CFU were predicted. The experiments performed by Rutschmann were carried out using colorimetric detection of PBP2x on Western blots. Here, we used chemiluminescent detection of PBP2x and two different methods to determined CFU of the R6 cultures used for lysate preparation. Both methods demonstrated similar results with PBP2x amounts, strongly suggesting the R6 cells contains an apparent surplus of PBP2x protein. In line with this, the depletion studies of wild-type PBP2x revealed that the strains can growth for at least four to five generations without detectable morphological defects [8]. Additional evidence that PBP2x is an abundant protein in the pneumococcal cell was provided by quantitative determination of PBP2x_C405_ present in the mutant strain C405. This strain contains drastically reduced amounts of PBP2x (16% or approximately 3200 molecules per cell) compared to the parental strain R6 (Figure 2), whereas growth and division was not significantly impaired at 30 °C with C405, demonstrating a slightly increased generation time (47 min) compared to parental strain R6 (42 min) (Figure S1). In other words, the amount of PBP2x in C405 was sufficient for proper growth. Since there are other alterations in the strain besides PBP2x mutations, it is not clear which mutation is associated with this phenotype. The importance of PBP2x is also apparent from several other studies. Kocaoglu et al. demonstrated that PBP2x is more sensitive against numerous β-lactam antibiotics than the other HMW PBPs [84]. The authors suggested that pneumococcal growth inhibition can be attributed primarily to the inhibition of PBP2x. Noirclerc-Savoye et al. found that the cellular amount of PBP2x was twofold less in comparison to PBP2b (720 ± 180) [83]. This contrasts with data obtained by Berg et al., where the depletion experiments indicated that PBP2b is needed in significantly smaller amounts than those required for PBP2x [7]. Until now, the cellular amount of very few cell division and elongation proteins in *S. pneumoniae* have been quantified [83,85,86]. In general, it is difficult to compare data between different studies, involving different strains, media, and experimental designs. It is important to compare the amounts of PBP2x protein to those of others cell division components and to elucidate the stoichiometry of these components.

In conclusion, our experiments suggest that the surface-exposed serine protease and putative chaperone HtrA targets PBP2x alleles that are structurally distinct from the wild-type PBP2x, thereby affecting PBP2x amounts in the cell, β-lactam resistance, cellular growth, and morphology. Further investigations such as the identification of the HtrA cleavage site in PBP2x could help to identify other cell surface proteins targeted by HtrA. A comprehensive HtrA target search will contribute to our understanding of the role of this important enzyme and to elucidate the function of CiaRH in the pneumococcal cell.

## Data Availability

All data are contained within the manuscript.

## Supplementary Materials

The following are available online at https://www.mdpi.com/article/10.3390/microorganisms9081685/s1: Figure S1: Characteristics of laboratory mutants C405 and C606. Figure S2. Impact of PBP2x mutations on growth and morphology. Figure S3: PBP2x and hyperactivated CiaRH system in R6*_2x_*_C606_ background. Figure S4: Nucleotide sequence of the P*_16S rRNA_* promoter region. Figure S5: Activity of promoters P*_htrA_*, P*_vegM_*, and P*_16S rRNA_* in *S. pneumoniae* R6 strain. Figure S6: Integration of pPP2 and pSW1 into *bgaA* region in *S. pneumoniae* R6 genome. Figure S7: Production of GFP-PBP2x_C405_ fusion protein in various genetic backgrounds. Table S1: *S. pneumoniae* strains used in this study. Table S2: Bacterial plasmids used in this study. Table S3: Primers used in this study.
