# Supplementary material for: New Insights into Beta-Lactam Resistance of Streptococcus pneumoniae: Serine Protease HtrA Degrades Altered Penicillin-Binding Protein 2x
Source: Microorganisms. 2021 Aug 8;9(8):1685. doi: 10.3390/microorganisms9081685 (PMC8400419; doi:10.3390/microorganisms9081685)
Supplement: Supplementary file 1 [file microorganisms-09-01685-s001.zip › microorganisms-1307039-Supplementary Material.pdf]

Supplementary materials

New insights into beta-lactam resistance of *Streptococcus pneumoniae*: serine protease HtrA degrades altered penicillin-binding protein 2x

Katharina Peters[¶, 1], Inga Schweizer[¶], Regine Hakenbeck[¶] and Dalia Denapaite[¶, 2,*]

[¶]Department of Microbiology, University of Kaiserslautern, Kaiserslautern, Germany

[1] present address: School of Natural & Environmental Sciences, Faculty of Science, Agriculture & Engineering, Newcastle University, Newcastle upon Tyne, NE1 7RU, UK

[2] present address: Department of Cellular, Computational and Integrative Biology, University of Trento, Trento, Italy

* Correspondence: dalia.denapaite@unitn.it

SUPPLEMENTAL TABLES

Table S1. *S. pneumoniae* strains used in this study

Table S2. Bacterial plasmids used in this study

Table S3. Primers used in this study

SUPPLEMENTAL EXPERIMENTAL PROCEDURES

SUPPLEMENTAL FIGURES

Figure S1

Figure S2

Figure S3

Figure S4

Figure S5

Figure S6

Figure S7

REFERENCES

SUPPLEMENTAL TABLES

Table S1. *S. pneumoniae* strains used in this study

Strain	Genotype or relevant feature[a, b]	Source or reference
R6	nonencapsulated derivative of the Rockefeller University strain R36A	Ottolenghi and Hotchkiss, 1962
C405	derivative of R6, genotype is partly unknown, *pbp2x* (L403F, T526S), *ciaH*305 (N95D); β-lactam^R	Laible und Hakenbeck, 1987
C606	derivative of R6, genotype is partly unknown, *pbp2x* (M289T, G422D, G597D, G601V), *ciaH*306 (T230P), *pbp2a* (S566Stop); β-lactam^R	Laible und Hakenbeck, 1987
KPKL5	C405, Δ*htrA::aphIII*; Kan^R	This study
KPKL51	KPKL5, *bgaA::tmp*-P$_{htrA}$-*htrA*$_{S234A}$; Tmp^R	This study
KPKL6	C606, Δ*htrA::aphIII*; Kan^R	This study
KPKL61	KPKL6, *bgaA::tmp*-P$_{htrA}$-*htrA*$_{S234A}$; Tmp^R	This study
R6$_{2xC405}$	R6, *pbp2x* (L403F, T526S); β-lactam^R	Maurer *et al.*, 2008
R6$_{2xC606}$	R6, *pbp2x* (M289T, G422D, G597D, G601V); β-lactam^R	Maurer *et al.*, 2008
R6$_{2xC405}$Δ*htrA*	R6$_{2xC405}$, Δ*htrA::aphIII*; Kan^R	This study
R6$_{2xC606}$Δ*htrA*	R6$_{2xC606}$, Δ*htrA::aphIII*; Kan^R	This study
R6^{strR}	R6, *rpsL41*; Str^R	Schweizer *et al.*, 2017
RKL161	R6, *rpsL41, ciaH::aphIII-rpsL*$^+$; Kan^R, Str^S	Müller *et al.*, 2011
KPKL20	R6$_{2xC405}$, *rpsL41*; Str^R	This study
KPKL201	R6$_{2xC405}$, *rpsL41, ciaH::aphIII-rpsL*$^+$; Kan^R, Str^S	This study
KPKL2011	R6$_{2xC405}$, *rpsL41, ciaH*305; Str^R	This study
KPKL10	R6$_{2xC606}$, *rpsL41*; Str^R	This study
KPKL101	R6$_{2xC606}$, *rpsL41, ciaH::aphIII-rpsL*$^+$; Kan^R, Str^S	This study
KPKL1011	R6$_{2xC606}$, *rpsL41, ciaH* (T230P, E255K); Str^R	This study
KPKL1012	R6$_{2xC606}$, *rpsL41, ciaH* (T230P, E255K); Str^R	This study
KPKL1013	R6$_{2xC606}$, *rpsL41, ciaH* (T230P), *ciaR* (G36T); Str^R	This study
RP200	R6, Δ*bgaA'::tetM-lacZ;* Tet^R	Halfmann *et al.*, 2007a
RKL43	R6, Δ*bgaA'::tetM*-P$_{htrA}$-*lacZ;* Tet^R	Halfmann *et al.*, 2007b
RKL45	R6, Δ*bgaA'::tetM*-P$_{vegM}$-*lacZ;* Tet^R	Halfmann *et al.*, 2007b
KPKL2	R6, Δ*bgaA'::tetM*-P$_{16S\ rRNA}$-*lacZ;* Tet^R	This study
KPKL1	R6, Δ*htrA::lox72*	This study
KPKL11	KPKL1, *bgaA::tmp*-P$_{htrA}$-*htr;* Tmp^R	This study
KPKL12	KPKL1, *bgaA::tmp*-P$_{vegM}$-*htrA;* Tmp^R	This study
KPKL131	KPKL1, *bgaA::tmp*-P$_{16S\ rRNA}$-*htrA*, ΔA 18 bases upstream of *htrA* starting codon, *htrA* (G703A at G235S (GGC→AGC)); Tmp^R	This study
KPKL132	KPKL1, *bgaA::tmp*-P$_{16S\ rRNA}$-*htrA*, ΔA 18 bases upstream of *htrA* starting codon; Tmp^R	This study
KPKL133	KPKL1, *bgaA::tmp*-P$_{16S\ rRNA}$-*htrA*, ΔG 9 bases upstream of *htrA* starting codon; Tmp^R	This study
KPKL134	KPKL1, *bgaA::tmp*-P$_{16S\ rRNA}$-*htrA*, ΔG 9 bases upstream of *htrA* starting codon; Tmp^R	This study
KPKL135	KPKL1, *bgaA::tmp*-P$_{16S\ rRNA}$-*htrA, htrA* (A361G at N121D (AAT→GAT)); Tmp^R	This study
KPKL14	KPKL1, *bgaA::tmp*-P$_{htrA}$-*htrA*$_{S234A}$; Tmp^R	This study
KPKL3	R6$_{2xC405}$, Δ*htrA::lox72*	This study
KPKL31	KPKL3, *bgaA::tmp*- P$_{htrA}$-*htrA;* Tmp^R	This study
KPKL321	KPKL3, *bgaA::tmp*-P$_{vegM}$-*htrA, htrA* (ΔT at F23L (TTT→TT-), S24V, G25E, A26P, L27W, G28V, S29V, S31Q, I32Stop); Tmp^R	This study

Table S1. *S. pneumoniae* strains used in this study (continued)

Strain	Genotype or relevant feature[a, b]	Source or reference
KPKL322	KPKL3, *bgaA*::*tmp*-P$_{vegM}$-*htrA*, *htrA* (ΩA at N283K (AAC→AAAC), G284R, V286S, T287D, R288A, P289S, A290S, L291T, I293N, Q294P, M295D, V296G, N297Stop); Tmp^R	This study
KPKL323	KPKL3, *bgaA*::*tmp*-P$_{vegM}$-*htrA*, *htrA* (A305G at E102G (GAA→GGA)); Tmp^R	This study
KPKL324	KPKL3, *bgaA*::*tmp*-P$_{vegM}$-*htrA*, *htrA* (T893C at L298S (TTA→TCA)); Tmp^R	This study
KPKL325	KPKL3, *bgaA*::*tmp*-P$_{vegM}$-*htrA*, *htrA* (ΔA at T6H (ACA→CAT), Y8T, K10N, W11G, Q13N, L14Y, L15Stop); Tmp^R	This study
KPKL4	R6$_{2xC606}$, Δ*htrA*::*lox72*	This study
KPKL41	KPKL4, *bgaA*::*tmp*-P$_{htrA}$-*htrA*; Tmp^R	This study
KPKL421	KPKL4, *bgaA*::*tmp*-P$_{vegM}$-*htrA*, *htrA* (T587C at V196A (GTC→GCC)); Tmp^R	This study
KPKL422	KPKL4, *bgaA*::*tmp*-P$_{vegM}$-*htrA*, *htrA* (T623A at V208A (GTA→GCA)); Tmp^R	This study
KPKL423	KPKL4, *bgaA*::*tmp*-P$_{vegM}$-*htrA*, *htrA* (T563C at L188S (TTA→TCA)); Tmp^R	This study
KPKL424	KPKL4, *bgaA*::*tmp*-P$_{vegM}$-*htrA*, *htrA* (A110G at Q37R (CAA→CGA)); T$_{-34}$C at P$_{vegM}$; Tmp^R	This study
KPKL425	KPKL4, *bgaA*::*tmp*-P$_{vegM}$-*htrA*, *htrA* (ΔA848 at N283T (AAC→-ACG), G284E, K285Stop), A$_{-99}$G at P$_{vegM}$; Tmp^R	This study
KPKL7	C405, Δ*bgaA'*::*tetM*-P$_{Zn}$-*gfp*-*pbp2x*$_{C405}$; Tet^R	This study
KPKL71	KPKL7, Δ*pbp2x*::*aad9*; Spc^R	This study
KPKL711	KPKL71, Δ*htrA*::*aphIII*; Kan^R	This study
KPKL8	R6$_{2xC405}$, Δ*bgaA'*::*tetM*-P$_{Zn}$-*gfp*-*pbp2x*$_{C405}$; Tet^R	This study
KPKL81	KPKL8, Δ*pbp2x*::*aad9*; Spc^R	This study
KPKL811	KPKL81, Δ*htrA*::*aphIII*; Kan^R	This study
DKL03	R6, Δ*bgaA'*::*tetM*-P$_{Zn}$-*gfp*-*pbp2x*; Tet^R	Peters *et al.*, 2014
DKL031	DKL03, Δ*pbp2x*::*aad9*; Spc^R	Peters *et al.*, 2014
DKL0311	DKL031, Δ*htrA*::*aphIII*; Kan^R	Peters *et al.*, 2014
DKL4	R6, Δ*bgaA*::*tetM*-P$_{Zn}$-*pbp2x*; Tet^R	Peters *et al.*, 2014
DKL41	DKL4, Δ*pbp2x*::*aad9*; Spc^R	Peters *et al.*, 2014
DKL41	DKL411, Δ*htrA*::*aphIII*; Kan^R	Peters *et al.*, 2014
DKL5	R6$_{2xC405}$, Δ*bgaA*::*tetM*-P$_{Zn}$-*pbp2x*$_{C405}$; Tet^R	This study
DKL51	DKL5, Δ*pbp2x*::*aad9*; Spc^R	This study
DKL511	DKL51, Δ*htrA*::*aphIII*; Kan^R	This study
DKL6	C405, Δ*bgaA'*::*tetM*-P$_{Zn}$-*pbp2x*$_{C405}$; Tet^R	This study
DKL61	DKL6, Δ*pbp2x*::*aad9*; Spc^R	This study
DKL611	DKL61, Δ*htrA*::*aphIII*; Kan^R	This study

[a]Strains were constructed as described in Supplemental experimental procedures. :: indicates an insertion into a region, Δ shows deletion into a region, Ω specifies an insertion; P- stands for promoter; P$_{Zn}$ stands for P$_{czcD}$, Zn^{2+} inducible promoter;

Antibiotic resistance markers: Tet, tetracycline; Kan, kanamycin; Spc, spectinomycin; Tmp, trimethoprim; Str, Streptomycin,

[b]The positions of aa in PBP2x gene are given according to GenBank accession number NC_003098.1. The positions of aa in HtrA gene are given according to locus_tag SPV_2068 in strain D39V (GenBank CP027540) [Slager *et al.*, 2018] since in the original annotation of *S. pneumoniae* R6 genome [Hoskins *et al.*, 2001] the start codon of *htrA* was wrongly assigned. The positions of aa in CiaR and CiaH genes are given according to locus_tags SPV_0701 and SPV_0702 respectively in strain D39V since in *S. pneumoniae* R6 genome the start codon of *ciaR* was wrongly assigned.

Table S2. Bacterial plasmids used in this study

Plasmid	Description[a]	Reference
p2xKO	pUC19 derivate [Yanish-Perron *et al.*, 1985] carrying an insert composed of upstream flanking region of *pbp2x*, spectinomycin resistance cassette and downstream flanking region of *pbp2x*; Amp^R	Peters *et al.*, 2014
pPP2[b]	Integrative promoter probe plasmid (*bgaA*::*tetM-lacZ*); Amp^R, Tet^R	Halfmann *et al.*, 2007a
pKL02	pPP2 derivative carrying $P_{16S\ rRNA}$-*lacZ* fusion; Amp^R, Tet^R	This study
pSW1	Integrative plasmid allowing integration of desired gene by double crossover at the *bgaA* locus in *S. pneumoniae* genome thereby replacing an intergenic region between *bgaA* and the adjacent gene spr0566; Amp^R, Tmp^R	Denapaite and Hakenbeck, 2011
pGEM-T Easy	TA cloning vector for PCR products	Promega
pKP1	pGEM-T-Easy derivate carrying HtrA gene with native P_{htrA} promoter; Amp^R	This study
pKP01	pSW1 derivative carrying HtrA gene with native P_{htrA} promoter; Amp^R, Tmp^R	This study
pJWV25	pPP2 derivative carrying P_{Zn}-*gfp*; Amp^R, Tet^R	Eberhardt *et al.*, 2009
pFP12	pJWV25 derivative carrying P_{Zn}-*gfp-pbp2x*$_{C405}$ fusion	This study
pFP13	pJWV25 derivative carrying P_{Zn}-*pbp2x*, (PBP2x 1–750 aa)	Peters *et al.*, 2014
pFP14	pJWV25 derivative carrying P_{Zn}-*pbp2x*$_{C405}$ fusion ($PBP2x_{C405}$ 1–750 aa)	This study
pQE-80L-Kan	Vector for expression of N-terminally 6 x His-tagged proteins; Kan^R	Qiagen
pKP041	pQE-80L-Kan derivative carrying His_6-$HtrA_{S234A}$oTM fusion, (HtrA amino acids residues 31-393[c]); $HtrA_{S234A}$ lacks proteolytic activity	This study
pPM20	pGEX-6P-1-tet derivative carrying GST-PBP2x (PBP2xWT 49-750aa), Tet^R	Maurer *et al.*, 2008

[a]Antibiotic resistance markers: Tet, tetracycline; Spc, spectinomycin; Tmp, trimethoprim; Amp, ampicillin; Kan, kanamycin.

[b]GenBank accession number EF061139.

[c]The positions of aa in *htrA* are given according to locus_tag SPV_2068 in strain D39V (GenBank CP027540) [Slager *et al.*, 2018] since in the original annotation of *S. pneumoniae* R6 genome [Hoskins *et al.*, 2001] the start codon of *htrA* was wrongly assigned.

Table S3. Primers used in this study

Name	Sequence (5′ to 3′)[a]	Used for construction of
16S_ppf	ACATGCATGCAGTCATGCAAAAAAAATG	pKP02, KPKL2
16S_ppr	GCGGGATCCGTTGCTATTTTCAACTCTTAC	pKP02, KPKL2
htrA_pcr1_f	CGTCCCAAATCCAAATAAATCCATTCTC	KPKL1, KPKL3, KPKL4
htrA_pcr1_r	GGGGACGCGTTTTCATATTTGCCTCCATATGTTTG	KPKL1, KPKL3, KPKL4
htrA_pcr3_f	GGGGCCATGGAATCTTAATTACATCTATGTAAAG	KPKL1, KPKL3, KPKL4
htrA_pcr3_r	AATCCACAATGTTACCAAACTTTATCC	KPKL1, KPKL3, KPKL4
Che_Mlu	GGGGACGCGTTGGCTTACCGTTCTATAGC	KPKL1, KPKL3, KPKL4
Cre_Nco	GGGGCCATGGTCGATACCGTTCTATAATG	KPKL1, KPKL3, KPKL4
htrA_kompl-NheI	CTAGCTAGCCTACACTAACACATCTTTTCTC	pKP01, pKP1, KPKL11, KPKL31, KPKL41
htrA_normal_f1	CGCGGATCCTCTCTATTTTTTCACATCTTATTC	pKP01, pKP1, KPKL11, KPKL31, KPKL41
pUK-M13 forward24	CGCCAGGGTTTTCCCAGTCACGAC	pKP1
pUK-M13 reverse22	TCACACAGGAAACAGCTATGAC	pKP1
IntFragA_up	TCATGACCGCGTTGACTATCTCAATGACGG	KPKL11, KPKL31, KPKL41
IntFragB_down	TAGCCCTCTCAATCCTAGCCATTATTCTCG	KPKL11, KPKL31, KPKL41

Table S3. Primers used in this study (continued)

Name	Sequence (5′ to 3′)[a]	Used for construction of
veg5	CGATGCATGCTTGGACTCCTGTTGATAGATCC	KPKL12, KPKL321-25, KPKL421-25
htrA_vegM_1	CTTATTAATATGATATAATTTGCAAAATTCAAACA TATGGAGGCAAATATG	KPKL12, KPKL321-25, KPKL421-25
htrA_vegM_2	CATATTTGCCTCCATATGTTTGAATTTTGCAAATT ATATCATATTAATAAG	KPKL12, KPKL321-25, KPKL421-25
htrA_16sRNA-P-1	AATAGTAAGAGTTGAAAATAGCAACAATTCAAA CATATGGAGGCAAATA	KPKL131-35
16sRNA_ppf_BamHI	CGCGGATCCAGTCATGCAAAAAAAATG	KPKL131-35
htrA-16sRNA-P_2	TATTTGCCTCCATATGTTTGAATTGTTGCTATTTTC AACTCTTACTATT	KPKL131-35
tet-htrA_f2	CGGGGTACCATGGAGGCAAATATGAAACATC	KPKL151, KPKL152
tet-htrA_r2	CACGCGGCTAGCTTATTAAGATTCTAAATCACCT GAAC	KPKL151, KPKL152
htrA_Ala_r	GCTATTAACCCAGGTAACCCGGCGGCCCACTGAT	KPKL14, KPKL51, KPKL61
htrA_Ala_f	ATCAGTGGGCCGCCGGCGTTACCTGGGTTAATAG C	KPKL14, KPKL51, KPKL61
rpsL_f	AGTGTACAGGGACGTGCTGACAAATGTTGC	KPKL20, KPKL10
rpsL_r	ATTGTATAGCGGATCTGGCAATACGTCACG	KPKL20, KPKL10
CiaHup_f	TGCAGTCGAAGCAGTTGTTCTTCAATACGG	KPKL201, KPKL101
CiaHdown_r	ATTGACCGCAACGAGCAATTCCTTACCAGC	KPKL201, KPKL101
CiaHup_ff	CGGTATGGGAATCAAGGAAATTGCAGCACG	KPKL2011, KPKL1011, KPKL1012, KPKL1013
CiaHdown_rr	TCCAAGAAGTCTAAGAAAGGCTTGATACGGTCC	KPKL2011, KPKL1011, KPKL1012, KPKL1013
pbp2x_gfp_r	ATAAGAATGCGGCCGCTTATTAGTCTCCTAAAGT TAATGTAATTTTTTTAATGTCC	pFP12
pbp2x_gfp_f	GCGCACTAGTAAGTGGACAAAAAGAGTAATCCG TTATGC	pFP12
2x-pFP14-f	TATGAAGTGGACAAAAAGAGTAATCCGTTATGCG	pFP14
pbp2x_gfp_r	ATAAGAATGCGGCCGCTTATTAGTCTCCTAAAGT TAATGTAATTTTTTTAATGTCC	pFP14
htrA_oTM_BamHI_f	CGCGGATCCATAACTCAACTAACTCAAAA AAGTAGTG	pKP41
htrA_overex_SalI_r	ACGCGTCGACTTAAGATTCTAAATCACCT GAACTCTTG	pKP41
RT-pbp2x-f	GACAGGGTTATTACTGGCGTTT	qRT-PCR
RT-pbp2x-r	GCTAACGACTAATACGTTGGAGA	qRT-PCR
RT-gyrAf	TATCACAGCAGTACGTGATGAG	qRT-PCR
RT-gyrAr	GGATAGCGAGCATATTGAAACC	qRT-PCR
RT-2045f	GTAGCCCGTTAGGTTCTGAATATG	qRT-PCR
RT-2045r	CCAGAGTTACCTGGGTTAATAGC	qRT-PCR

[a]The red underlined text in the primer sequence column indicates restriction sites.

SUPPLEMENTAL EXPERIMENTAL PROCEDURES

Recombinant DNA techniques and oligonucleotides

Standard protocols for molecular cloning, transformation, and DNA analysis were used [Sambrook *et al.*, 1989]. The oligonucleotides were purchased from Eurofins Genomics and are listed in Table S3. The plasmid DNA purification from *E. coli* was done according to the manufacturer's protocol using the QIAprep Spin Miniprep kit (Qiagen). Genomic DNA was isolated from *S. pneumoniae* as described earlier [Laible *et al*., 1989]. PCR products and DNA recovered after restriction endonuclease digestions were purified using a JETquick DNA Purification kit (Genomed) or Nucleo-Spin Extract II kit (Macherey-Nagel). Restriction enzymes and T4 DNA ligase were purchased from Fermentas (Thermo Fisher Scientific), Invitrogen or New England Biolabs and used as described by the manufacturer. DNA fragments from agarose gels were purified by using innuPREP Gel Extraction Kit (Analytik Jena). PCRs were performed using either RedGoldStar Taq polymerase (Eurogentec), DreamTaq™-Polymerase (Thermo Fisher Scientific), *Pfu*-Polymerase (New England BioLabs) or iProof high fidelity DNA polymerase (Bio-Rad Laboratories) according to the manufacturer's instructions.

Plasmid and strain constructions

All constructed plasmids were verified by DNA sequencing. Correct integration of the plasmids or linear amplicon via double crossover in the pneumococcal genome was confirmed by PCR and DNA sequencing of the chromosomal region corresponding to the amplicon or plasmid and surrounding regions.

Construction of promoter probe-based plasmid and strain

To construct the plasmid pKP02, the promoter region ($P_{16S\ rRNA}$) of *S. pneumoniae* R6 16S rRNA gene was amplified from strain R6 using the primer pair 16S_ppf/16S_ppr. The resulting PCR product was digested with SphI and BamHI and ligated into pPP2 [Halfmann *et al*., 2007a] digested with the same enzymes. The resulting plasmid was named pKP02. The plasmid was transformed to *S. pneumoniae* R6 strain using the tetracycline resistance marker *tetM* for selection as described previously [Halfmann *et al*., 2007a]. The resulting strain was named KPKL2.

Construction of htrA deletion strains using the kanamycin resistance cassette

The *htrA* gene was deleted in different strains by replacement with the kanamycin resistance cassette as described previously [Peters *et al*., 2014].

Construction of htrA deletion strains using the Cheshire cassette

For marker-less deletion of the *htrA* gene in *S. pneumoniae* a self-excising mutagenic Cre-Lox construct known as Cheshire cassette was used [Weng *et al*., 2009]. Up and downstream fragments of *htrA* were amplified using the primer pairs htrA_pcr1_f/htrA_pcr1_r and htrA_pcr3_f and htrA_pcr3_r (Table S3). Genomic DNA of *S. pneumoniae* R6 was used as template for both PCRs. The fragments were digested with restriction enzymes MluI and NcoI. A Cheshire cassette (Genbank accession No. FJ981645) carrying the erythromycin-resistance marker (*ermAM*), which can be used as a temporary marker for selection, was kindly provided by Donald Morrison (University of Illinois at Chicago) and used as template in the PCR with the primers Che_Mlu/Cre_Nco. The resulting PCR products were digested with restriction enzymes MluI and NcoI. All three amplicons were agarose gel-purified and ligated into a linear DNA fragment. The ligation mix was directly transformed into *S. pneumoniae* R6, $R6_{2xC405}$ and $R6_{2xC606}$ strains and transformants were selected with 0.1 µg/ml erythromycin. Subsequently, an *htrA* deletion strain harbouring *lox72* in place of *htrA* was obtained by the published procedure [Weng *et al*., 2009]. The fidelity of Cheshire excision was confirmed by PCR and sequencing. The resulting strains were named KPKL1, KPKL3 and KPKL4.

Construction of HtrA complementation strains

To complement deletion of HtrA gene in three different strains KPKL1, KPKL3 and KPKL4 and to achieve ectopic expression of *htrA* gene under the control of different promoters, a series of integrative

plasmids based on pSW1 plasmid [Denapaite and Hakenbeck, 2011] were constructed. Plasmid pSW1 carries the flanking regions homologous to *S. pneumoniae* sequences allowing integration into the chromosome by double crossover at the *bgaA* locus thereby replacing an intergenic region between *bgaA* and the adjacent gene spr0566 and leaving the possibility for integration of promoter probe-based plasmid [Halfmann *et al.,* 2007a] (Figure S5). The integrative part of pSW1 is stably maintained in the genome of *S. pneumoniae* and no further selection is needed. Plasmid pSW1 carries a trimethoprim resistance marker, which can be used for selection of the transformants in *S. pneumoniae*, and the β-lactamase gene (*bla*) confers ampicillin resistance in *E. coli*.

Complementation of *htrA* under the control of native promoter. To achieve ectopic expression of *htrA* under the control of native promoter, plasmid pKP01 was constructed. The sequence encoding *htrA* under the control of native promoter was amplified with the primer pair htrA_kompl-NheI/htrA_normal_f1 (Table S3) and with the RedGoldStar Taq polymerase using genomic DNA of *S. pneumoniae* R6 strain as template. The PCR product was subsequently cloned into the vector pGEM-T Easy vector according to the manufacturer's instructions and standard techniques. The constructed plasmid, named pKP1, was confirmed by PCR amplification with the primer pair pUK-M13 forward24/pUK-M13 reverse22 as well as DNA sequencing (Table S3). The plasmid pKP1 was cleaved with BamHI and NheI, the fragment carrying *htrA* was ligated into the corresponding sites of pSW1. The constructed plasmid was verified by sequencing and named pKP01. Subsequently the plasmid pKP01 was transformed into *S. pneumoniae* strains KPKL1, KPKL3 and KPKL4. Positive transformants were selected on plates containing trimethoprim. The correct integration between *bgaA* and spr0566 was confirmed by PCR using primer pair IntFragA_up und IntFragB_down and the introduced *htrA* gene and promoter region were again sequenced. Using this strategy the complementation strains KPKL11, KPKL31 and KPKL41 were constructed.

Complementation of *htrA* under the control of *vegM* promoter. To achieve ectopic expression of *htrA* under the control of constitutive promoter *vegM* (P_{vegM}), a PCR was performed using primers veg5 and htrA_vegM_2 and genomic DNA from *S. pneumoniae* RKL45 [Halfmann *et al*., 2007b] as a template. The primers htrA_vegM_1 and htrA_kompl-NheI were used to amplify *htrA* gene from genomic DNA of *S. pneumoniae* R6 strain. Both fragments were gel-purified and joined by overlapping PCR using the primer pair veg5/htrA_kompl-NheI. The resulting fragment was purified from agarose gel, cleaved with restriction enzymes BamHI and NheI and ligated into the corresponding sites of pSW1. When the ligation mixture was transformed into *E. coli* DH5α strain, no transformants were obtained. Therefore, this ligation mixture was directly transformed into *S. pneumoniae* KPKL1, KPKL3 and KLKP4 strains. Transformants of the strain KPKL1 were sequence verified, and the strain was named KPKL12 (Table S1). The transformants of another two strains (KPKL3 and KLKP4), which contain an altered *pbp2x* gene, were confirmed by PCR using a primer pair IntFragA_up/IntFragB_down. The complementation strains were designed KPKL321-KPKL325 and KPKL421-KPKL425, respectively (Table S1). The sequencing of HtrA gene fused with P_{vegM} promoter region of these transformants showed different mutations either in *htrA* gene (transformants of strain KPKL3) or both in *htrA* gene and in promoter region of *htrA* gene (transformants of strain KPKL4). The mutations are listed in Table S1.

Complementation of *htrA* under the control of 16S rRNA promoter. To achieve ectopic expression of *htrA* under the control of the very strong constitutive promoter $P_{16S\ rRNA}$, the promoter region of the 16S rRNA gene was fused with *htrA* by overlapping PCR. The *htrA* gene was amplified by PCR without its native promoter using the primer pair htrA_kompl-NheI und htrA_16sRNA-P-1, whereas the promoter region $P_{16S\ rRNA}$ was amplified using the primer pair 16sRNA_ppf_BamHI und htrA-16sRNA-P_2 (Table S3). Genomic DNA of *S. pneumoniae* R6 was used as a template for both PCRs. The PCR products were gel-purified and used as templates in a subsequent PCR using the outer primers 16sRNA_ppf_BamHI and htrA_kompl-NheI. The resulting PCR product was gel-purified, digested with BamHI and NheI, and ligated into the corresponding sites of pSW1. Initially the ligation mix was transformed into *E. coli* DH5α cells, but colonies on the transformation plates were transparent and died after transfer to new

plates. Therefore, the ligation mixture was directly used for transformation of strain KPKL1 selecting for clones resistant to trimethoprim. The transformants were confirmed by PCR using a primers IntFragA_up und IntFragB_down. The complementation strains were designed KPKL131-KPKL135 (Table S1). Sequencing the region of the $P_{16S\ rRNA}$ *htrA* fusion of the transformants KPKL131-KPKL135 revealed different mutations either in *htrA* gene or in promoter region of *htrA* gene. The mutations are listed in Table S1.

Construction of HtrA$_{S234A}$ strains using the site directed mutagenesis

To construct strains containing the S_{234}A mutation in HtrA at the catalytic site, site-directed mutagenesis was used to produce the mutated *htrA*. The mutagenesis resulted in a change of the Ser codon TCT at position 712 (position 234 at aa level) of the *htrA* DNA sequence to the Ala codon GCC, thereby inactivating proteolytic activity of HtrA. Notably, the codon optimization was performed according Prokaryotes Codon Usage Database (http://exon.gatech.edu/GeneMark/metagenome/CodonUsageDatabase/). The mutated *htrA* gene was designated *htrA$_{S234A}$*. Mutagenesis was performed by an overlapping PCR approach using primer pairs htrA_normal_f1/htrA_Ala_f and htrA_kompl-NheI/htrA_Ala_r and genomic DNA of strain R6 as a template. Primers htrA_Ala_f and htrA_Ala_r introduced the desired mutation. Two resulting amplicons were gel-purified and used as templates in a following PCR using the primer pair htrA_normal_f1 and htrA_kompl-NheI. The resulting PCR product was digested with *BamH*I and *Nhe*I and ligated into plasmid pSW1 [Denapaite and Hakenbeck, 2011] digested with the same enzymes. The ligation mixture was directly transformed into *S. pneumoniae* cells using the trimethoprim resistance marker for selection. Correct integration into the genome was verified by PCR. KPKL14, KPKL51 and KPKL61 (Table S1) strains were generated by the same procedure.

Introduction of ciaH alleles into S. pneumoniae *R6$_{2xC405}$ and R6$_{2xC606}$ strains*

The Janus counter selection procedure [Sung *et al.*, 2001] was chosen as the strategy to replace the *ciaH* wild-type R6 allele with *ciaH305* and *ciaH306* variants in the two strains $R6_{2xC405}$ and $R6_{2xC606}$ as described previously [Müller *et al*., 2011]. First, the *rpsL41* allele was amplified using primer pair rpsL-f/rpsL-r and genomic DNA of $R6^{strR}$ strain as template and introduced into R62*x*C405 and R62*x*C606 strains. The strains were designated KPKL20 and KPKL10, respectively. In a second step, the *ciaR*::Janus (*aphIII-rpsL*$^{+}$) fragment was amplified using primer pair CiaHup_f/CiaHdown_r and genomic DNA of RKL161 as a template. The resulting fragment was transformed into the streptomycin resistant strains KPKL20 and KPKL10 carrying the *rpsL41* allele. Streptomycin-sensitive, kanamycin-resistant transformants (named as KPKL201 and KPKL101) were then used as the recipient for the new *ciaH* alleles. Genomic DNA from the laboratory mutants C405 and C606 was used for the amplification of *ciaH305* and *ciaH306* by PCR using primers CiaHup_ff and CiaHdown_rr. The *ciaH* PCR fragments were introduced into KPKL201 and KPKL101 strains by selecting streptomycin-resistant transformants. Subsequently, the *ciaRH* region of these strains was sequenced. One transformant, KPKL2011, which carries *ciaH305* allele, was used for all further experiments. The sequencing results of three transformants, which carries *ciaH306* allele, showed the additional mutations either in *ciaH* or in *ciaR* genes. The strains were designated KPKL1011, KPKL1012 and KPKL1013 (Table S1).

Construction of strains expressing GFP-PBP2x$_{C405}$ fusion protein

To construct the plasmid pFP12 carrying the *gfp*+ variant fused to PBP2x gene from laboratory mutant strain C405 under the control of the zinc-inducible P_{Zn} promoter, a PCR was performed using the primer pair pbp2x_gfp_r/pbp2x_gfp_f and genomic DNA of C405 laboratory mutant strain as a template. The PCR product subsequently was cleaved with *Not*I and *Spe*I and ligated into the corresponding sites of vector pJWV25 [Eberhardt *et al.*, 2009], generating the plasmid pFP12.

The plasmid pFP12 was transformed into *S. pneumoniae* C405 and $R6_{2xC405}$ strains selecting for clones resistant to tetracycline. Transformants were verified for correct insertion of the respective P_{Zn}-*gfp*-$2x_{C405}$

construct at the *bgaA* locus by PCR and the mutations in *pbp2x* by DNA sequencing. The merodiploid strains were named KPKL7 and KPKL8, respectively.
The *pbp2x* gene was deleted in the KPKL7 und KPKL8 genetic background in the presence of Zn with the help of plasmid p2xKO (Table S2) containing the spectinomycin resistance gene *aad9* as well as the flanking regions of *pbp2x* as described previously [Peters *et al.*, 2014; Schweizer *et al.*, 2014]. The conditional mutants of *pbp2x* were named KPKL71 und KPKL81, respectively.

Construction of pbp2x *conditional mutants*
To construct plasmid pFP14 which expresses *pbp2x*$_{C405}$ allele from the zinc inducible promoter P$_{Zn}$, the plasmid pFP13 was digested with NgoMIV and NotI and the resulting 8765 bp fragment was gel purified. The *pbp2x*$_{C405}$ gene was amplified using the primer pair 2x-pFP14-f/*pbp2x*_gfp_r and plasmid DNA of pFP12 as a template. The resulting PCR product was digested with restriction enzymes NgoMIV and NotI and ligated with 8765 bp fragment to yield the plasmid pFP14.
To construct the merodiploid strains which contain two copies of *pbp2x*$_{C405}$: the genomic *pbp2x*$_{C405}$ which is expressed constitutively [Peters *et al.*, 2016], and the zinc-inducible ectopic *pbp2x*$_{C405}$, plasmid pFP14 was transformed into *S. pneumoniae* C405 and R6$_{2xC405}$ strains followed by the selection of clones resistant to tetracycline. Transformants were screened by PCR and the presence of mutations in PBP2x gene was confirmed by DNA sequencing. The strains were named DKL5 and DKL6, respectively.
To construct conditional mutant of *pbp2x*, the PBP2x gene was deleted in the DKL5 and DKL6 genetic background using the plasmid p2xKO containing the spectinomycin resistance gene in the presence of Zn as described previously [Peters *et al.*, 2014; Schweizer *et al.*, 2014]. The conditional mutants of *pbp2x* were named DKL51 und DKL61, respectively.

Construction of pKP041 plasmid
The pneumococcal *htrA*$_{S234A}$ gene lacking the signal sequence [SPV_2068 in strain D39V (GenBank CP027540) (spr2045 in R6 genome), nt 94-1182 corresponding to amino acid residues 31-393] carrying a mutation at the putative catalytic site was amplified by PCR using primers htrA_oTM_BamHI_f and htrA_overex_SalI_r. *S. pneumoniae* KPKL51 genomic DNA was used as template. Both primers had incorporated restriction sites BamHI and SalI, respectively (Table S3). The amplified fragment *htrA*$_{S234A}$oTM (oTM stands for without signal sequence) was purified, double digested with BamHI/SalI, re-purified and ligated into the BamHI/SalI digested pQE-80L-Kan vector (Table S2). The ligation mixture was transformed in *E. coli* DH5α competent cells and transformants were selected using the kanamycin resistance marker. The resulting plasmid was verified by PCR and DNA sequencing of the insert and named pKP041.

Figure S1. Characteristics of laboratory mutants C405 and C606

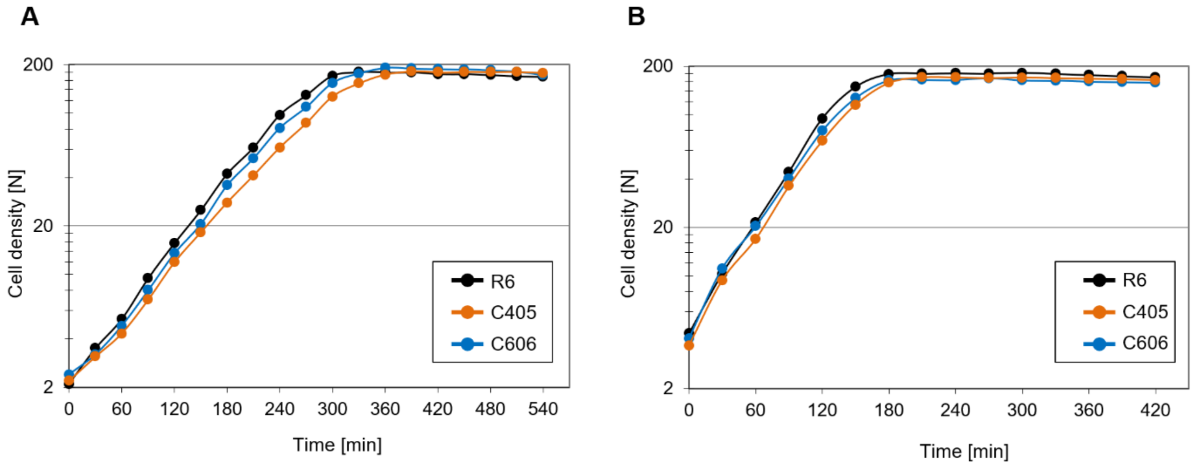

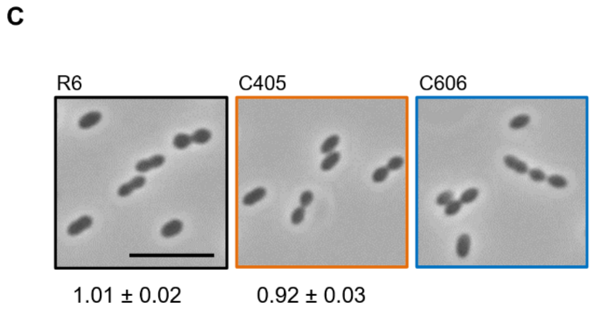

D

Strain	Doubling time (min)	
	30ºC	37ºC
R6	42	28
C405	47	31
C606	43	28

Figure S1. Characteristics of laboratory mutants C405 and C606.
Growth curves of *S. pneumoniae* mutants C405 and C606 in C+Y medium in comparison to parental strain R6. (**A**) Growth at 30°C (**B**) Growth at 37°C. Cellular growth was monitored by nephelometry (N, nephelometry units). (**C**) Phase-contrast micrographs of C405, C606 and R6 cells grown at 30°C. Scale bar, 5µm. C405 mutant cells at the mid-exponential growth phase were smaller (0.92 µm ± 0.03, (± indicates the standard deviation), n=174 cells) in comparison to the cell length of R6 (1.01 µm ± 0.02, n=180 cells). Due to genetic instability the cells of C606 strain were not measured. (**D**) Doubling times of C405, C606 and R6 strains were measured at two different temperatures.

Figure S2. Impact of PBP2x mutations on growth and morphology

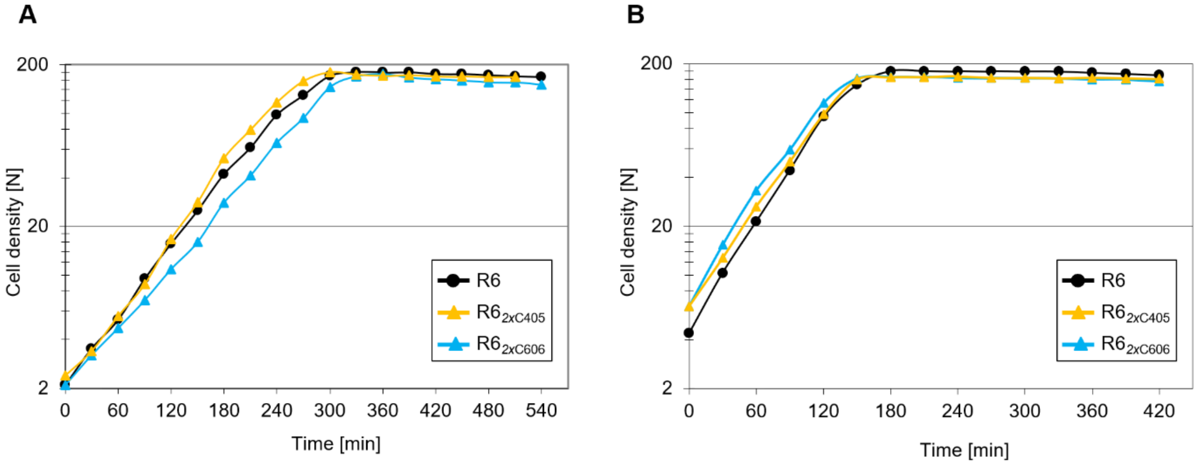

C

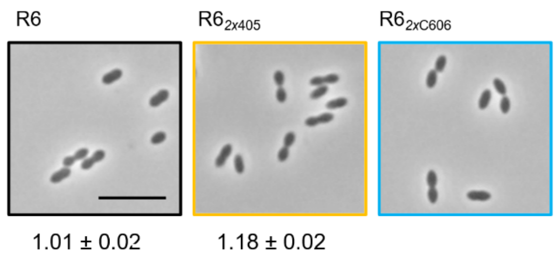

D

Strain	Doubling time (min) 30ºC	37ºC
R6	42	28
R6$_{2xC405}$	41	31
R6$_{2xC606}$	48	32

Figure S2. Impact of PBP2x mutations on growth and morphology.
Growth curves of *S. pneumoniae* R6$_{2xC405}$ and R6$_{2xC606}$ in C+Y medium in comparison to parental strain R6. (**A**) Growth at 30°C (**B**) Growth at 37°C. Cellular growth was monitored by nephelometry (N, nephelometry units). (**C**) Representative phase-contrast micrographs of the R6$_{2xC405}$, R6$_{2xC606}$ and R6 cells grown at 30°C. Scale bar, 5µm. The cells of R6$_{2xC405}$ were slightly longer (1.18m ± 0.02, n=86 cells) in comparison to the cell length of R6 (1.01 µm ± 0.02, n=180 cells). (**D**) Doubling times of R6$_{2xC405}$, R6$_{2xC606}$ and R6 strains were measured at two different temperatures.

Figure S3. PBP2x and hyperactivated CiaRH system in R6$_{2xC606}$ background

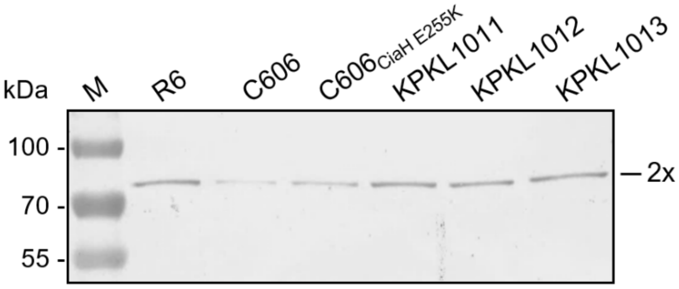

Figure S3. PBP2x and hyperactivated CiaRH system in R6$_{2xC606}$ background.
Western blot analysis using anti-PBP2x antibodies. Strains used for preparation of cell lysates are indicated on top. Strains: KPKL1011 and KPKL1012 (R6, PBP2x M289T, G422D, G597D, G601V, CiaH T230P, E255K), KPKL1013 (R6, PBP2x M289T, G422D, G597D, G601V, CiaH T230P, CiaR G36T). M, Protein marker II (PeqLab). The strain C606 *ciaH* E255K contains an additional mutation in *ciaH* gene which may directly affects and downregulate the activity of CiaR-dependent promoters.

Figure S4. Nucleotide sequence of the P$_{16S\ rRNA}$ promoter region

```
 SphI                                        -35
...GCATGCAGTCATGCAAAAAAAAATGAAAAAAAATTAGAAAAAGTAGTTGACAAAGTTTGAAAAG

    -10                          BamHI            SD
GCTGTATAATAGTAAGAGTTGAAAATAGCAACGGATCCTAACTAGTTAAGGAGGCAAATATGAAACATCTTGAT
                                                            >>M  K  H  L  D
```

Figure S4. Nucleotide sequence of the P$_{16S\ rRNA}$ promoter region.
The promoter was cloned as SphI-BamHI (in grey) fragment (underlined). The -35 region is marked in orange, the -10 region is shown in green, and the Shine-Dalgarno (SD) [Shine and Dalgarno, 1974] sequence is highlighted in red. The start codon of *lacZ* gene is indicated in red.

Figure S5. Activity of promoters P_{htrA}, P_{vegM} and $P_{16S\ rRNA}$ in *S. pneumoniae* R6 strain

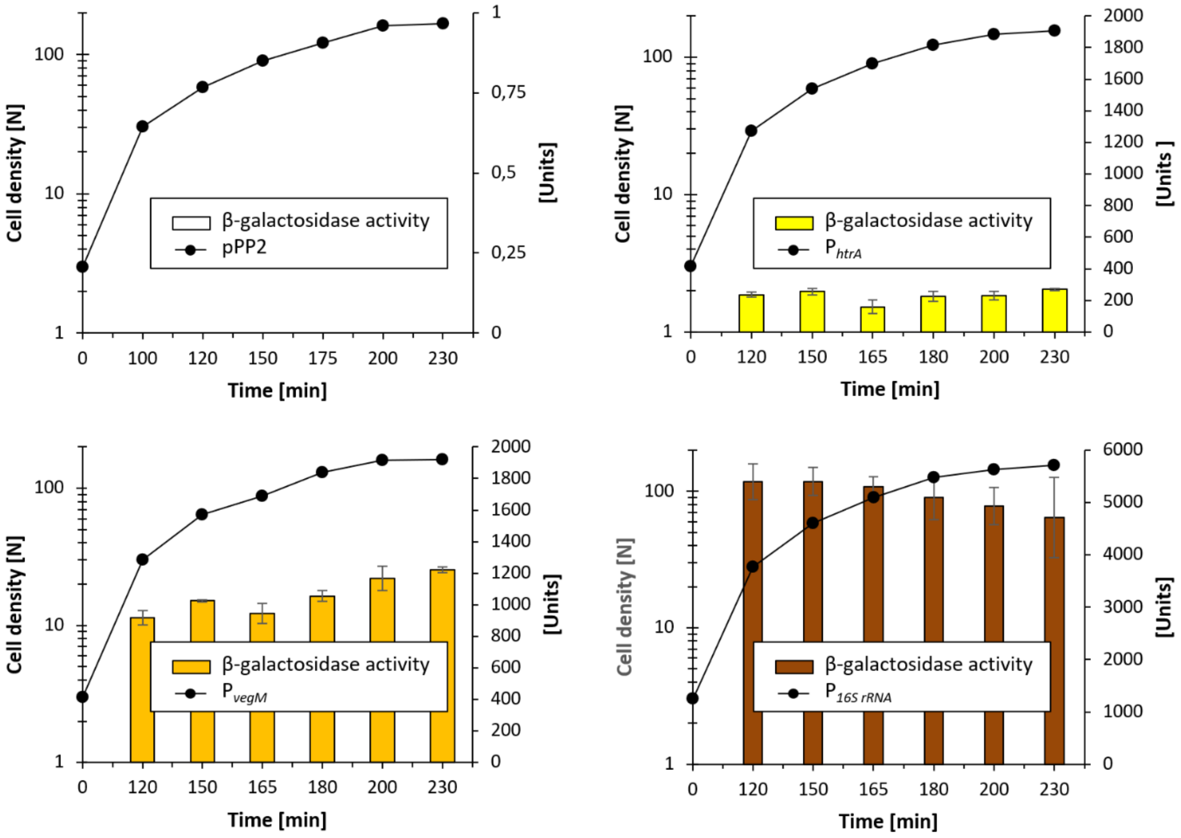

Figure S5. Activity of promoters P_{htrA}, P_{vegM} and $P_{16S\ rRNA}$ in *S. pneumoniae* R6.
Strains were grown in C+Y medium. Growth curves of one representative experiment are shown. The promoter activities were determined by measuring beta-galactosidase activities in strains carrying P_{htrA}, P_{vegM} and $P_{16S\ rRNA}$ promoter fragments cloned in front of a promoterless beta-galactosidase. pPP2 (empty plasmid) was used as a control. Beta-galactosidase activities were measured throughout the growth of the culture at six time points indicated. Beta-galactosidase units are given in nanomoles of nitrophenol released per minute and milligram of protein. Mean values and standard deviations from two independent experiments are presented. The beta-galactosidase activities are shown at different scales.

The native promoter of HtrA, which is a moderate promoter and strongly dependent on CiaR [Halfmann *et al.*, 2007b], showed an activity of approximately 200 miller units during growth in C+Y medium. The constitutive expressed *vegM* (P_{vegM}) belongs to the strong promoters and correspond to the HtrA expression level in C606 mutant strain. A new cloned promoter $P_{16S\ rRNA}$ mediated a very high level of beta-galactosidase activity of 5-time higher than P_{vegM} promoter.

Figure S6. Integration of pPP2 and pSW1 into *bgaA* region in *S. pneumoniae* R6 genome

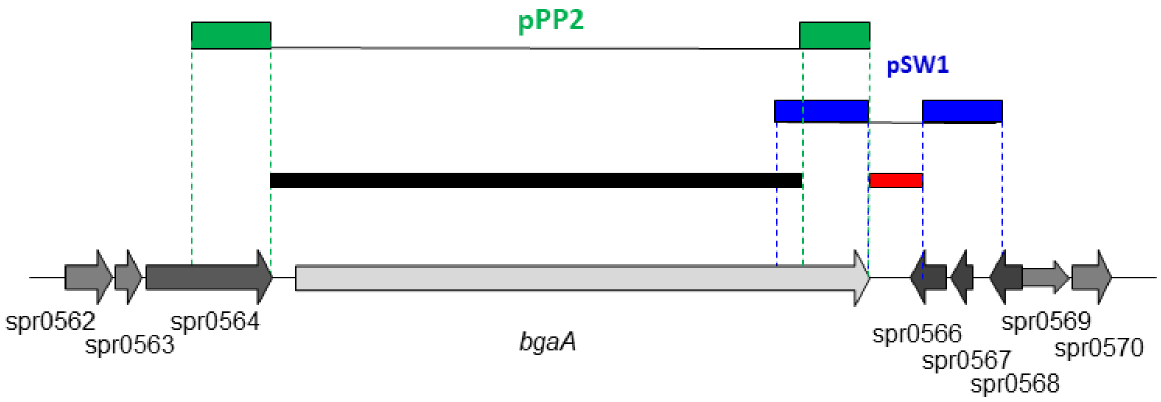

Figure S6. Integration of pPP2 and pSW1 into *bgaA* region in *S. pneumoniae* R6 genome.
The *bgaA* region in *S. pneumoniae* R6 genome is shown. Two different plasmids pPP2 [Halfmann *et al.*, 2007a] and pSW1 [Denapaite and Hakenbeck, 2011] integrate at the different places in the *bgaA* locus by a double crossover event, which leads to stable integration. Upon integration of the pPP2 plasmid, the endogenous *bgaA* gene is disrupted (shown in black and this part of *bgaA* will be replaced with genes carrying by pPP2 plasmid). The homology regions between pPP2 and R6 genome are shown in green. The integration of pSW1 plasmid into R6 genome replaces the intergenic region between *bgaA* gene and the adjacent gene spr0566 (shown in red) with genes carrying by pSW1 plasmid. The homology regions between pSW1 and R6 genome are shown in blue.

The simultaneous use of those two plasmids in experiment allows the ectopic integration of the gene of interest in *S. pneumoniae* genome and at the same time to use promotor probe plasmid pPP2.

Figure S7. Production of GFP-PBP2x$_{C405}$ fusion protein in various genetic backgrounds

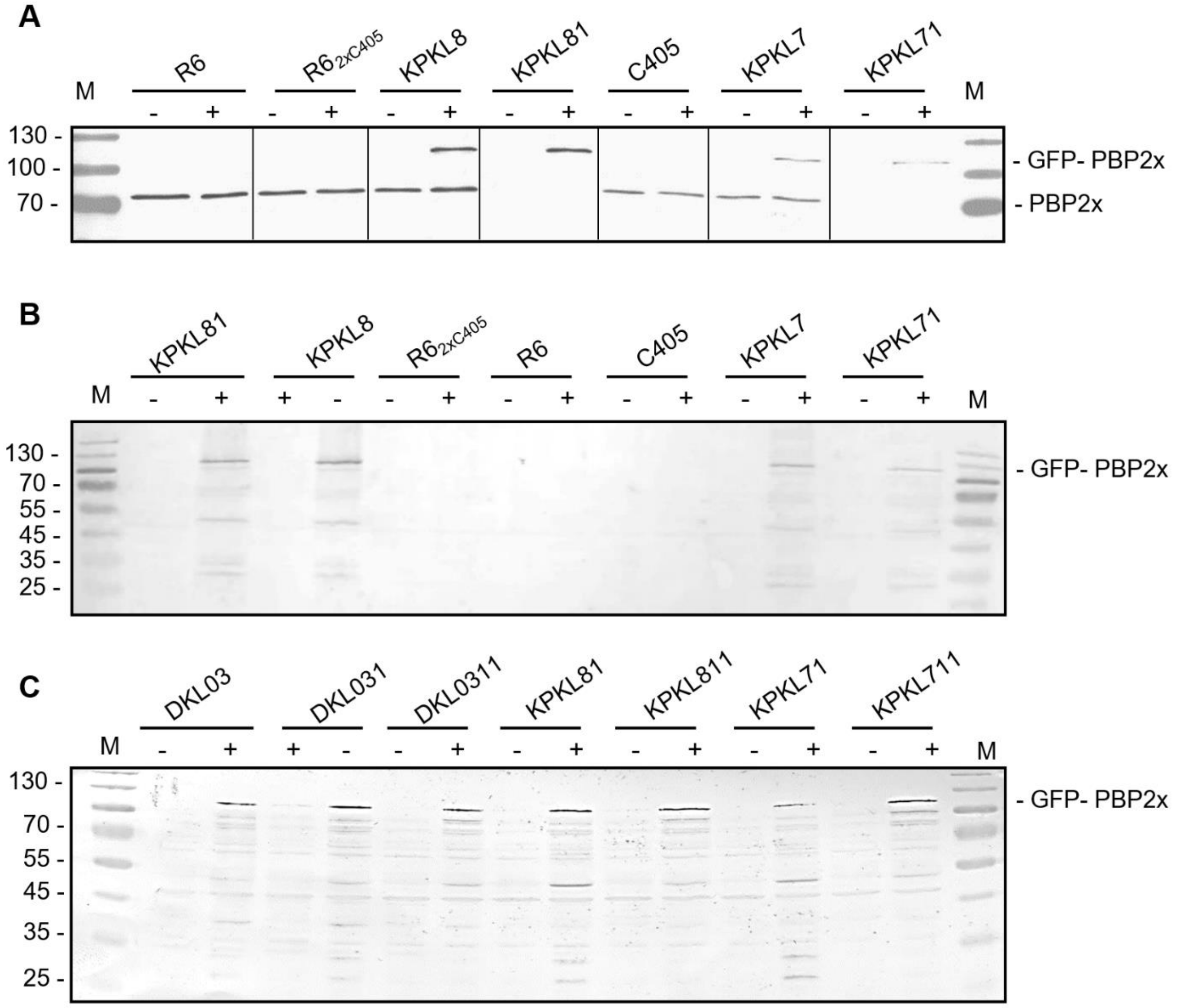

Figure S7. Production of GFP-PBP2x$_{C405}$ fusion protein in various genetic backgrounds.
(**A**) Western blot analysis using anti-PBP2x antibodies. (**B**) Western blot of cell lysates developed with anti-GFP antibodies. (**C**) Effect of *htrA* deletion on production of GFP-PBP2x$_{C405}$ fusion protein. Western blot developed with anti-GFP (α-GFP) antibodies. Strains used for preparation of cell lysates are indicated on top. The positions of GFP-PBP2x and PBP2x are indicated on the right. Cells were grown in the absence (-) or presence (+) of 0.15 mM $ZnCl_2$. Strains: KPKL7, C405, P$_{Zn}$-*gfp-2x*$_{C405}$; KPKL71, KPKL7 Δ*2x*$_{C405}$; KPKL711, KPKL71 *ΔhtrA*; KPKL8, R6$_{2xC405}$ P$_{Zn}$-*gfp-2x*$_{C405}$; KPKL81, KPKL8 Δ*2x*$_{C405}$; KPKL811, KPKL81 *ΔhtrA*; DKL03, R6 P$_{Zn}$-*gfp-2x*; DKL031, DKL03 Δ*2x*; DKL0311, DKL031 *ΔhtrA*.

REFERENCES

1. Ottolenghi, E.; Hotchkiss, R.D. Release of genetic transforming agent from pneumococcal cultures during growth and disintegration. *J Exp Med* **1962**, *116*, 491–519. DOI: 10.1084/jem.116.4.491
2. Laible, G.; Hakenbeck, R. Penicillin-binding proteins in β-lactam-resistant laboratory mutants of *Streptococcus pneumoniae*. *Mol Microbiol* **1987,** *1*, 355–363. DOI: 10.1111/j.1365-2958.1987.tb01942.x
3. Maurer, P.; Koch, B.; Zerfaß, I.; Krauß, J.; van der Linden, M.; Frère, J.-M., Contreras-Martel, C.; Hakenbeck, R. Penicillin-binding protein 2x of *Streptococcus pneumoniae:* three new mutational pathways for remodelling an essential enzyme into a resistance determinant. *J Mol Biol* **2008**, *376*, 1403-1416. DOI: 10.1016/j.jmb.2007.12.058
4. Schweizer, I.; Blättner, S.; Maurer, P.; Peters, K.; Vollmer, D.; Vollmer, W.; Hakenbeck, R.; Denapaite, D. New aspects of the interplay between penicillin binding proteins, *murM*, and the two-component system CiaRH of penicillin-resistant *Streptococcus pneumoniae* serotype 19A isolates from Hungary. *Antimicrob Agents Chemother* **2017**, *61*, e00414-17. DOI: 10.1128/AAC.00414-17
5. Müller, M.; Marx, P.; Hakenbeck, R.; Brückner, R. Effect of new alleles of the histidine kinase gene *ciaH* on the activity of the response regulator CiaR in *Streptococcus pneumoniae* R6. *Microbiology* **2011,** *157*, 3104-3112. DOI: 10.1099/mic.0.053157-0
6. Halfmann, A.; Hakenbeck, R.; Brückner, R. A new integrative reporter plasmid for *Streptococcus pneumoniae*. *FEMS Microbiol Lett* **2007a**, *268*, 217–224. DOI: 10.1111/j.1574-6968.2006.00584.x
7. Halfmann, A.; Kovács, M.; Hakenbeck, R.; Brückner, R. Identification of the genes directly controlled by the response regulator CiaR in *Streptococcus pneumoniae*: five out of fifteen promoters drive expression of small noncoding RNAs. *Mol Microbiol* **2007b**, *66*, 110–126. DOI: 10.1111/j.1365-2958.2007.05900.x
8. Peters, K.; Schweizer, I.; Beilharz, K.; Stahlmann, C.; Veening, J.W.; Hakenbeck, R.; Denapaite. D. *Streptococcus pneumoniae* PBP2x mid-cell localization requires the C-terminal PASTA domains and is essential for cell shape maintenance. *Mol Microbiol* **2014**, *92*, 733-755. DOI: 10.1111/mmi.12588
9. Slager, J.; Aprianto, R.; Veening, J.W. Deep genome annotation of the opportunistic human pathogen *Streptococcus pneumoniae* D39. *Nucleic Acids Res* **2018**, *46*, 9971-9989. DOI: 10.1093/nar/gky725
10. Hoskins, J.; *et al.*, Genome of the bacterium *Streptococcus pneumoniae* strain R6. *J Bacteriol* **2001**, *183*, 5709-5717. DOI: 10.1128/JB.183.19.5709-5717.2001
11. Yanish-Perron, C.; Vieira, J.; Messing, J. Improved M13 phage cloning vectors and host strains: nucleotide sequence of the M13mp18 and pCU19 vectors. *Gene* **1985**, *33*, 103–119. DOI: 10.1016/0378-1119(85)90120-9
12. Denapaite, D.; Hakenbeck, R. A new variant of the capsule 3 cluster occurs in *Streptococcus pneumoniae* from deceased wild chimpanzees. *PLoS One* **2011**, *6*, e25119. DOI: 10.1371/journal.pone.0025119
13. Eberhardt, A.; Wu, L.J.; Errington, J.; Vollmer, W.; Veening, J.W.; Cellular localization of choline utilization proteins in *Streptococcus pneumoniae* using novel fluorescent reporter systems. *Mol Microbiol* **2009**, *74*, 395-408. DOI: 10.1111/j.1365-2958.2009.06872.x
14. Sambrook, J.; Fritsch, E.F.; Maniatis, T. Molecular cloning: a laboratory manual, 2nd ed.; Cold Spring Harbor Laboratory Press: Cold Spring Harbor, NY, USA, 1989.
15. Laible, G.; Hakenbeck, R.; Sicard, M.A.; Joris, B.; Ghuysen, J.-M. Nucleotide sequences of the *pbpX* genes encoding the penicillin-binding protein 2x from *Streptococcus pneumoniae* R6 and a cefotaxime-resistant mutant, C506. *Mol Microbiol* **1989**, *3*, 1337-1348. DOI: 10.1111/j.1365-2958.1989.tb00115.x
16. Weng, L.; Biswas, I.; Morrison, D.A. A self-deleting Cre-lox-ermAM cassette, Cheshire, for marker-less gene deletion in *Streptococcus pneumoniae*. *J Microbiol Methods* **2009**, *79*, 353-357. DOI: 10.1016/j.mimet.2009.10.007
17. Sung, C.K.; Li, H.; Claverys, J.P.; Morrison, D.A. An *rpsL* cassette, Janus, for gene replacement through negative selection in *Streptococcus pneumoniae*. *Appl Environ Microbiol* **2001**, *67*, 5190-5196. DOI: 10.1128/AEM.67.11.5190-5196.2001
18. Schweizer, I.; Peters, K.; Stahlmann, C.; Hakenbeck, R.; Denapaite, D. Penicillin-binding protein 2x of *Streptococcus pneumoniae*: the mutation Ala707Asp within the C-terminal PASTA2 domain leads to destabilization. *Microb Drug Resist* **2014**, *20*, 250-257. DOI: 10.1089/mdr.2014.0082
19. Peters, K.; Pipo, J.; Schweizer, I.; Hakenbeck, R.; Denapaite, D. Promoter Identification and Transcription Analysis of Penicillin-Binding Protein Genes in *Streptococcus pneumoniae* R6. *Microb Drug Resist* **2016**, 22, 487-498. DOI: 10.1089/mdr.2016.0084
20. Shine, J.; Dalgano L. The 3'-terminal sequence of *Escherichia coli* 16S ribosomal RNA: complementarity to nonsense triplets and ribosome binding sites. Proc Natl Acad Sci USA **1974**, 71, 1342-1346. DOI: 10.1073/pnas.71.4.1342
